# A revision of the *Encarsia mexicana* species-group (=*Dirphys* Howard) (Hymenoptera: Aphelinidae), gregarious endoparasitoids of whiteflies (Hemiptera: Aleyrodidae) in the Neotropical Region †

**DOI:** 10.3390/insects14060570

**Published:** 2023-06-20

**Authors:** Andrew Polaszek, Estrella Hernández-Suárez, Robert L. Kresslein, Paul Hanson, Yvonne M. Linton, Jacqueline MacKenzie-Dodds, Stefan Schmidt

**Affiliations:** 1 Insects Division, Natural History Museum, London SW7 5BD, UK; 2 Unidad Protección Vegetal, Instituto Canario de Investigaciones Agrarias, 38270 San Cristóbal de La Laguna, Spain; 3 Department of Entomology, University of California, Riverside, CA 92521, USA; 4 Escuela de Biología, Universidad de Costa Rica, San Jose 11501-2060, Costa Rica; 5 Smithsonian Institution, Museum Support Center, Walter Reed Biosystematics Unit, Suitland, MD 20746, USA; 6 Molecular Collections Facility, Natural History Museum, London SW7 5BD, UK; 7 SNSB-Zoologische Staatssammlung München, 81247 Munich, Germany

**Keywords:** Aleyrodidae, Aleurodicinae, parasitoid, biological control, new world

## Abstract

**Simple Summary:**

*Encarsia* (Family Aphelinidae) is a genus of minute parasitoid wasps that target a diversity of agricultural pest insects including whiteflies and armoured scale insects. Since the genus was described in 1878, 450+ species of *Encarsia* have been described. Historically, it has been difficult to provide a subgeneric classification for the species of *Encarsia*. As a result, researchers have divided the group into numerous informal species-groups. Our work uses an alignment of ribosomal DNA sequences (sequences that code for ribosomal RNA) to construct a phylogenetic tree that shows that species of the genus *Dirphys* are correctly placed within *Encarsia*. With these results, we establish the *Encarsia mexicana* species-group (for the six species previously placed in *Dirphys*) and describe 14 new species. We also briefly discuss morphological characters that may correspond to the relationships recovered in the molecular phylogeny. With this information, we can better understand the patterns of evolution which brought about the present diversity within *Encarsia* and provide a more accurate classification of the genus.

**Abstract:**

The genus *Dirphys* Howard 1914 **syn. n.** is synonymized with *Encarsia* Förster, and treated as a species-group of *Encarsia*, referred to henceforth as the *Encarsia mexicana* species-group. The monophyly of *Encarsia* is discussed in relation to *Dirphys*. The new synonymy is based on phylogenetic analyses of the nuclear ribosomal 28S-D2 gene region (43 taxa, 510 bp). The *Encarsia mexicana* species-group is recovered as strongly monophyletic within *Encarsia.* All species of the *Encarsia mexicana* species-group are revised. The group includes six previously described species, and fourteen newly described species. All species are described (or redescribed) and illustrated. Detailed distributional data, and, where available, plant associate and host records are provided for all species. *Encarsia myartsevae* Kresslein and Polaszek **nom. nov.** is here proposed as a replacement name for *Encarsia mexicana* Myartseva, now preoccupied by *Encarsia mexicana* (Howard). A dichotomous identification key, supplemented by an online multiple-entry key, is provided for all species.

## 1. Introduction

The genus *Dirphys* was initially described by Howard [1] for *Mesidia mexicana* Howard [2]. Where known, species in this genus are primary endoparasitoids of Aleyrodidae [3,4,5] and are gregarious, with up to 16 developing in a single host [3,4]. This behavior is unknown in any other chalcid parasitoids of whiteflies. *Dirphys* has been regarded as occupying a transitional zone between *Coccophagus* Westwood and *Encarsia* Förster [3] due to its having intermediate characters of those genera, especially with regard to setation of the mesoscutal mid lobe—more setose than most *Encarsia*, less setose than most *Coccophagus*. However, it displays a unique morphological synapomorphy of the sculpture of the dorsal mesosoma, which is always markedly rugose, irrespective of whether the pattern is aciculate (Figure 6E and Figure 11E), transverse (Figure 10E) or longitudinal (Figure 4C and Figure 20E), or contains combined elements of these patterns (Figure 12E and Figure 13E). Importantly, reticulate mesosomal sculpture is unknown in *Dirphys*. A second apparent autapomorphy concerns the division of the mesoscutal side lobes (see e.g., Figure 4C, Figure 6E, Figure 18E and Figure 23E). These apparent autapomorphies notwithstanding, analyses of the 28S-D2 ribosomal DNA (Kresslein et al. unpublished), and loci recovered with Anchored Hybrid Enrichment (Cruaud et al. unpublished, Kresslein et al. unpublished) show *Dirphys* nested within an otherwise monophyletic *Encarsia*. Further confusion about the relationship between *Dirphys* and *Encarsia* arose with the description of *Encarsiella* Hayat [6], which bears a strong superficial resemblance to *Dirphys* and was at one time synonymized with it [7,8]. *Encarsiella* was synonymized with *Encarsia* by Shafee and Rizvi [9] and is here regarded as the *Encarsia noyesi* species-group [10,11].

A preliminary study into phylogenetic relationships within the subfamily Coccophaginae was undertaken by Polaszek and Hayat [3] based on 24 morphological characters. In that work, the monophyly of *Dirphys* was supported by a single synapomorphy, the mesoscutal and scutellar sculpture. Another character supporting the monophyly of *Dirphys* was the proximity of the scutellar sensilla, although this character was known to have evolved independently many times within *Encarsia* [12]. In the same work, Polaszek and Hayat revised the species of *Dirphys* known at that time, describing three new species, *D. diablejo* Polaszek and Hayat, *D. encantadora* Polaszek and Hayat, and *D. mendesi* Polaszek and Hayat. Chavez [4] described a fifth species, *D. larensis* Chavez from Venezuela, and Polaszek added a sixth, *D. aphania* Polaszek [5].

In the present manuscript, we synonymize *Dirphys* (hereinafter referred to as the *mexicana* species-group) with *Encarsia*. Using maximum likelihood analysis of 28s D2 rDNA (43 taxa, 510 bp), we recover the *Encarsia mexicana* species-group as strongly monophyletic within *Encarsia.* We provide a comprehensive revision of the known species of the *Encarsia mexicana* species-group with species description (or redescription), illustrations, distributional data, and, where available, plant associate and host records. Fourteen species are described here as new: *Encarsia acusa* Polaszek and Hernández-Suárez **sp. n.***, Encarsia aisha* Polaszek and Hernández-Suárez **sp. n.***, Encarsia avida* Polaszek and Hernández-Suárez **sp. n.***, Encarsia catula* Polaszek and Hernández-Suárez **sp. n.***, Encarsia cylindrica* Polaszek and Hernández-Suárez **sp. n.***, Encarsia dichaeta* Polaszek and Hernández-Suárez **sp. n.***, Encarsia erwini* Polaszek and Hernández-Suárez **sp. n.***, Encarsia fredbennetti* Polaszek and Hernández-Suárez **sp. n.***, Encarsia inbioa* Polaszek and Hernández-Suárez **sp. n.***, Encarsia napo* Polaszek and Hernández-Suárez **sp. n.***, Encarsia marynoyesae* Polaszek and Hernández-Suárez **sp. n.***, Encarsia noora* Polaszek and Hernández-Suárez **sp. n.***, Encarsia svetlana* Polaszek and Hernández-Suárez **sp. n.***,* and *Encarsia venia* Polaszek and Hernández-Suárez **sp. n.** The name, *Encarsia myartsevae* Kresslein and Polaszek **nom. nov.** is here proposed as a replacement name for *Encarsia mexicana* Myartseva [13], now preoccupied by *Encarsia mexicana* (Howard). We also provide a dichotomous identification key, supplemented by an online multiple-entry key for all species of the *Encarsia mexicana* species-group.

## 2. Materials and Methods

### 2.1. Specimen Depositories: Abbreviations

Material examined as a part of this investigation is deposited at the following institutions.

NHMUK: Natural History Museum, London, UK.

UCRC: University of California, Riverside, USA.

USNM: National Museum of Natural History, Smithsonian, Washington D.C., USA.

MZUCR: Museo de Zoología Universidad de Costa Rica.

### 2.2. Morphological Study

Populations of the *Encarsia mexicana* species-group were studied from different localities (Table 1). Host-reared material was collected in Costa Rica, Ecuador, Mexico, and Trinidad and Tobago, as part of intensive foreign exploration efforts to search for parasitoids of whitefly pests (Hemiptera: Aleyrodidae), mostly in the subfamily Aleurodicinae. For morphological analysis, female specimens were mounted on microscope slides following Noyes [14] with some modifications as follows: no maceration in 10% KOH was needed after DNA extraction. Specimens were washed in distilled water for one hour and then dehydrated for 5 min in graded ethanol of the following concentrations: 35%, 70%, 85%, and 100%. After clearing in clove oil and allowing alcohol evaporation, specimens were dissected in Canada balsam. The wings, antennae, head, and remaining body parts were mounted separately on a single slide.

**Figure 1:**
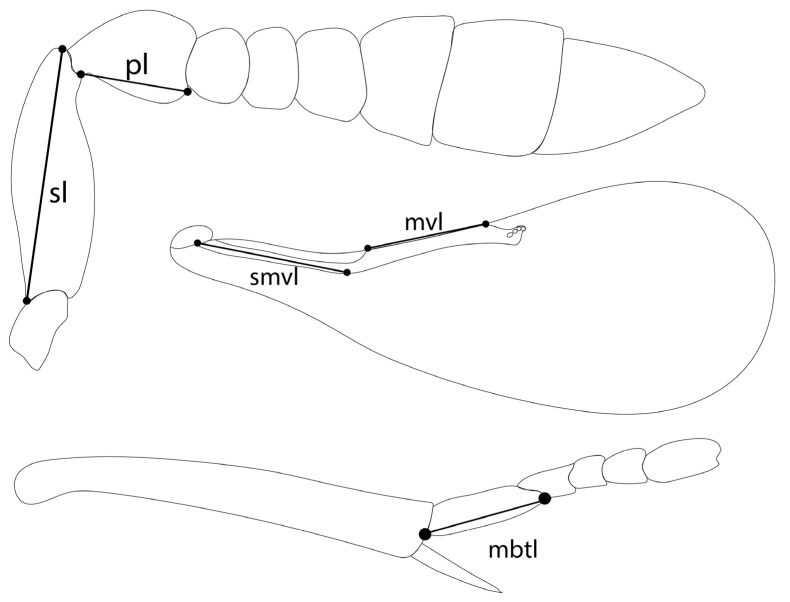
Additional measurements used in the present study: scape length (sl); pedicel length (pl); submarginal vein length (smvl); marginal vein length (mvl); mid basitarsus length (mbtl). Illustrated from type material of *Encarsia aisha*.

In total, 110 females and 4 males of 20 species were examined, including the extensive recording of measurements and ratios. Males are rare or unknown for most species and were not therefore included, except for *Encarsia diablejo* which is known only from the male. Measurements were taken with a Leitz Dialux 20EB microscope from slide-mounted material following Heraty and Polaszek [12] with the following five measurements added: scape length, pedicel length, submarginal vein length, marginal vein length and length of the mid basitarsus. (Figure 1). All measurements of antennae, fore wings, and legs refer to the maximum length of the structure in lateral view. The terminology of morphological characters follows Kim and Heraty [15] and Hayat [16].

Specimens were imaged using a Leitz Dialux 20EB (Wetzlar, Germany) compound microscope using Nomarski Differential Interference Contrast illumination (DIC) and photographed with a MicroPublisher 5.0 RTV (QImaging, Surrey, Canada) camera. Additional images (claval sensorial area; mandibles) were imaged with an Olympus BX63 microscope (Olympus, Tokyo, Japan) also utilizing DIC. Scanned sections were stacked and combined using Synoptics AutoMontage Pro^®^ ver. 5.03 software (Leitz Dialux images) and Helicon Focus software (Olympus BX63 images). The final images were edited with Adobe Photoshop CC^®^.

### 2.3. DNA Extraction, Amplification, and Sequencing

Genomic DNA was extracted from single, whole specimens using a non-destructive genomic DNA extraction protocol developed by Chao-Dong Zhu, John Noyes, and others at the Natural History Museum, London [17]. Occasionally 2–3 specimens were extracted together when known to be conspecific. 

Specimens were softened in 70% ethanol (to reduce potential damage during subsequent steps) at room temperature for a minimum of 2 h. Seventy percent ethanol was removed carefully by pipette and specimens were allowed to air-dry briefly. DNA was extracted using the Qiagen DNeasy Blood and Tissue Kit (250) #69506 (Qiagen, Hilden, Germany). Specimens were immersed in 180 µL of Lysis Buffer ATL, premixed with 20 µL Proteinase K and incubated at 55 °C overnight (8 h minimum) with no mixing, taking care that the specimen was submerged/floating in the buffer and not adhered to the side of the tube.

After digestion, the lysis buffer was carefully removed by pipette into a clean 1.5 mL microfuge tube. The specimen was immediately washed by adding 500 µL distilled water for a minimum of 30 min, then replaced with 500 µL 70% ethanol for a minimum of 30 min, then finally stored in 100% ethanol until slide-mounted in Canada balsam.

DNA was extracted from the lysis buffer using the Qiagen QUIA quick PCR Purification Kit (250) #28106 following the protocol: ‘Isolation of total DNA from Animal Tissues’ (step 3 onwards). Standard PCR reactions were then carried out in a thermal cycler using 2.0 µL DNA extract, Taq buffer (1.5 mM MgCl2), 1.5 U Taq polymerase (Roche), 10 nmol dNTPs (Amersham Pharmacia Biotech; APB) and 20 mol of each primer at the Natural History Museum’s DNA sequencing facility.

The D2 region of 28S rDNA was amplified using the following primers: 

28SFW 5′-AGTACCGTGAGGGAAAGTTG-3′

28SRev 5′-TTGGTCCGTGTTTCAAGACGG 3′

PCR conditions were as follows: an initial denaturation of 94 °C for 3 min, then 35 cycles of denaturation at 94 °C for 1 min, annealing at 50 °C for 1 min, and extension at 72 °C for 2 min, followed by a final extension at 75 °C for 10 min, then samples were held at 4 °C until they could be analyzed. PCR products were run on a 1% agarose gel to confirm PCR success (clean bands of the expected size), then the remaining products were cleaned and sequenced. Removal of dye terminators was done by ethanol precipitation prior to sequencing. 

The DNA analyzer system ABI PRISM 3730 and 377 DNA sequencer were used, the samples were loaded onto the system’s vertical polyacrylamide gel where they underwent electrophoresis, laser detection and computer analysis. Sequence editing and alignment were performed using Sequencher TM 4.8 (Gene Codes Corporation, Ann Arbor, USA) on a Macintosh computer. The resulting molecular dataset includes 18 sequences representing 13 species. Sequences have been deposited in the GenBank database under accession numbers OQ683545–OQ683576 (Table 1 and Table 2).

### 2.4. Phylogenetic Analyses

Captured sequences were combined with previously published sequence data (Table 2) and aligned using the E-INS-I algorithm in MAFFT v7.490 [18]. Ten independent iterations of maximum likelihood were reconstructed using IQ-TREE version 2.0.7 [19], implementing a General Time Reversible model with invariant sites and gamma distributed rate variation (-m GTR + I + G). Bootstrap support was estimated from 1000 bootstrap trees constructed using ultrafast bootstrapping (-b 1000) [20]. Outgroups are comprised of a broad range of *Encarsia* species, a diversity of recognized species-groups, as well as *Coccophagus* Westwood (Aphelinidae: Coccophaginae) and *Aphytis* Howard (Aphelinidae: Aphelininae).

### 2.5. Nomenclatural Acts

The electronic edition of this article conforms to the requirements of the amended International Code of Zoological Nomenclature, and hence the new names contained herein are available under that Code from the electronic edition of this article. This published work and the nomenclatural acts it contains have been registered in ZooBank, the online registration system for ICZN. The ZooBank LSIDs (Life Science Identifiers) can be resolved, and the associated information can be viewed through any standard web browser by appending the LSID to the prefix “http://zoobank.org/ (accessed on 6 June 2023)”. The LSID for this publication is urn:lsid:zoobank.org:pub:2CE58923-A39A-412A-896E-DCFD4CC01FD7. The electronic edition of this work was published in a journal with an ISSN and has been archived and is available from the following digital repositories: PubMed Central, LOCKSS.

## 3. Results

### 3.1. Phylogenetic Analysis of Molecular Data

**Figure 2:**
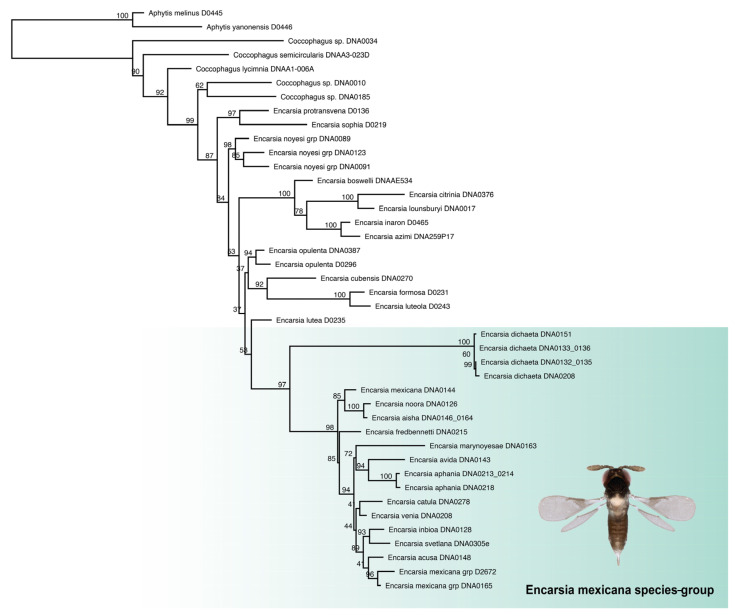
Maximum likelihood tree (IQ-TREE 2) based on 28S D2 ribosomal DNA (509 bp) from 42 taxa (19 ingroups, 23 outgroups); support values from 1000 ultrafast bootstrap replicates.

A maximum likelihood tree was constructed from partial sequences of 28S D2 ribosomal DNA of 13 species (from 18 specimens) and 23 outgroup taxa (Figure 2). The *Encarsia mexicana* species-group was recovered as a strongly supported clade within *Encarsia*. *Encarsia dichaeta* forms the sister clade to all remaining *E. mexicana*-group species. The *Encarsia mexicana* species-group was not placed sister to the *noyesi* species-group; however, backbone support in the recovered phylogeny is insufficient to confidently resolve inter- and intra-species-group relationships.

### 3.2. Taxonomy of the Encarsia mexicana Species-Group

***Encarsia mexicana* species-group**

**= *Dirphys* Howard, 1914**

Etymology of *Dirphys*. *Dirphys* (Διρφυς) is a Greek feminine noun. Hence the modification by Hayat of Howard’s (1914) combination *Dirphys mexicana* (Howard) to *Dirphys mexicanus* (Howard) was an unjustified emendation [21]. Hayat attributed the new combination to Howard [1], but this is not the case.

Diagnosis: head dorsally transverse. Frontovertex at narrowest wider than dorsal eye width. Facial lines evident, often broadly expanded; mediofrontal and transfacial lines developed. Eyes with evident setae. Mandibles usually with two teeth and a truncation (Figure 10A), the truncation sometimes reduced, and the teeth often strongly developed so mandibles appear to have only two teeth (Figure 22A). A bidentate upper tooth may be present in addition to the well-developed ventral tooth (Figure 4A).

Maxillary palps two-segmented. Antenna eight-segmented in both sexes, antennal formula variable (1, 1, 3, 3 or 1, 1, 0, 6), claval sensorial complex present (Figure 17B) or absent, suture between F5 perpendicular or oblique. Pronotum medially membranous. Mesoscutum with more than 20 setae. Side lobes divided (Figure 23E). Axillae large, strongly projecting forwards and separated medially by less than the maximum length of one axilla. Each axilla usually with a single seta, in *E. dichaeta* with two setae (Figure 11E). Thoracic sculpture aciculate, longitudinal, transverse or a combination of these types, never reticulate, always strongly rugose. Mesoscutellar sensilla close together, separated by about the width of one sensillum. Fore wings with two large setae on the submarginal vein, plus a variable number of smaller setae at the distal end of the submarginal vein. Linea calva present or absent. Mid basitarsus with a variable number of robust, spine-like setae, tarsi five-segmented.

**Figure 3:**
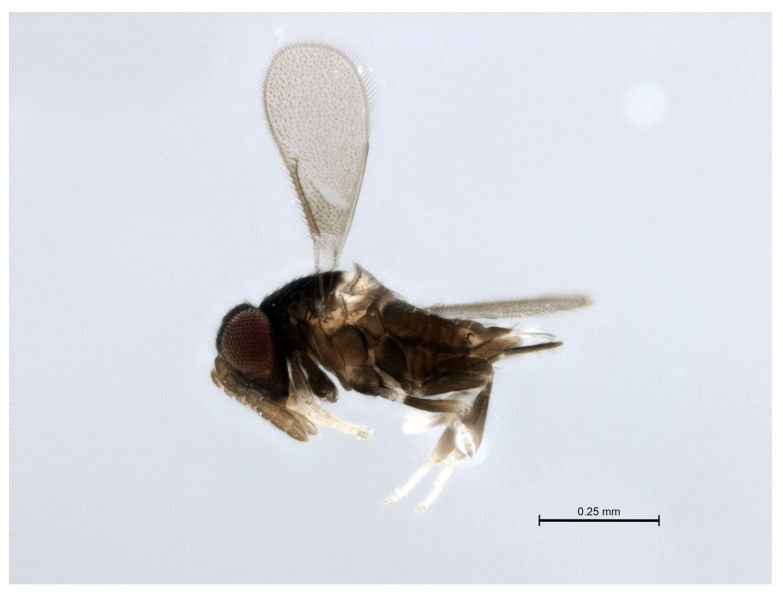
Lateral habitus of a female of the *Encarsia mexicana* species-group.

Remarks: The *Encarsia mexicana* species-group (Figure 3) is restricted to the Neotropical zone, with species reaching as far south as the State of Bahia (Brazil), and as far north as southern Mexico.

### 3.3. Species Descriptions

#### 3.3.1. *Encarsia acusa* Polaszek and Hernández-Suárez **sp. n.**

(Figure 4A–F)

urn:lsid:zoobank.org:act:F179717C-0FF0-4189-90BE-4851A8625431

Female. Colour: Antennae light brown; radicle, scape, base of pedicel and F6 darker. Head dark brown, paler along the sutures and frons. Mesosoma and metasoma dark brown with posterior 80% of the mesoscutellum and sides of metanotum yellow. Legs yellow with most of the mid and hind femora brown, fore femora and tibiae brown, all tarsi pale. Fore wings hyaline, slightly infuscate below marginal vein, submarginal and marginal veins dark. 

**Figure 4:**
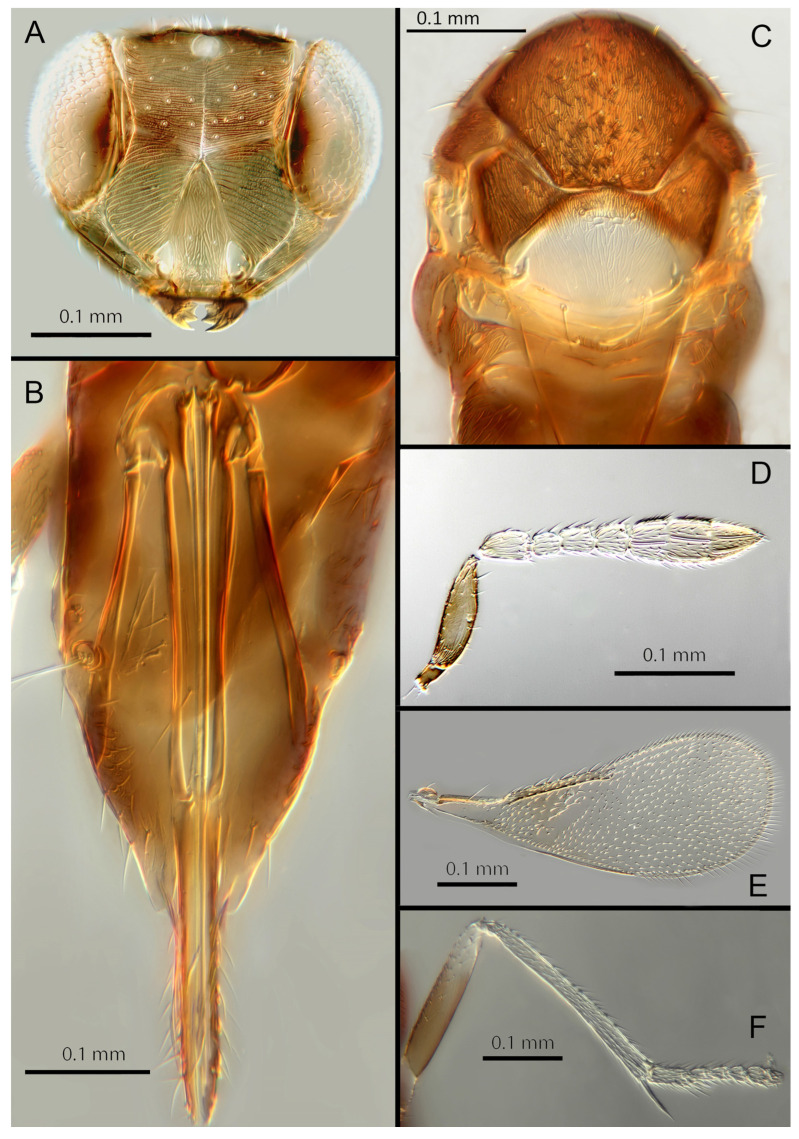
***Encarsia acusa***: (**A**) head; (**B**) ovipositor; (**C**) dorsal mesosoma; (**D**) antenna; (**E**) fore wing; (**F**) mid leg.

Morphology: Head (Figure 4A) with mediofrontal line complete; transfacial line obscure; facial lines narrow. Scrobes with longitudinal aciculate sculpture. Antennal formula (Figure 4D): 1, 1, 3, 3; scape 2.4x pedicel length; pedicel 1.9x F1; F1 0.8x F2; F2 equal to F3; funicle 0.56x clava; F6 slightly oblique, claval sensorial area present. Flagellum with the following number of longitudinal sensilla: F1: 0; F2: 1; F3: 1; F4: 3; F5: 4; F6: 3–4 (both counts present in holotype). Mandibles (Figure 4A) with two teeth and a broad truncation. Maxillary palps two-segmented. Mid-lobe of mesoscutum (Figure 4C) with 34–40 setae; each lateral lobe with two setae; each axilla with one seta; scutellum with four setae. Sculpture of mesoscutum longitudinal; sculpture of axillae and scutellum longitudinal. Fore wing (Figure 4E) with two large setae and 2–3 smaller setae on the submarginal vein, three setae in the basal cell, 6–7 setae on the anterior margin of the marginal vein, and one seta at the junction of the submarginal vein and parastigma. Linea calva present. Submarginal 0.75x marginal vein. Maximum length of fore wing 2.7x fore wing width; maximum width of wing 5.3x longest seta on marginal fringe. Ovipositor (Figure 4B) 1.85x mid tibial length; third valvulae 0.44x ovipositor length; second valvifer 1.3x third valvula. Mid tibial spur (Figure 4F) 0.86x corresponding basitarsus. Metasomal terga T1–T7 with 0, 2 + 2, 2 + 2, 1 + 1, 1 + 2 + 1, 1 + 2 + 1 and four setae, respectively. T7 (Figure 4B) extremely extended, almost covering the ovipositor.

Distribution. COSTA RICA: Heredia, Limon; PERU: Iquitos.

Material examined: Holotype ♀ COSTA RICA, Heredia Estación Biológica La Selva, 75 m, 10°26′ N 84°01′ W 27–28.ii.2003 (J.S. Noyes) [DNA148: OQ683554] (NHMUK). Paratypes: 1♀ COSTA RICA, Limon RB, Hitoy-Cerere 100 m, 14–19.i.1991 (J.S. Noyes) (MZUCR). 1♀ PERU, Iquitos, Barillal, 10.ii.1984 (L. Huggert #BM 1984.337) [DNA 212] (NHMUK).

Remarks: T7 extremely extended, covering the ovipositor. *Encarsia acusa* appears to be most closely related to *E. inbioa* and *E. svetlana*, but is easily distinguished from those (and all other) species by the extremely long ovipositor and T7. DNA sequences from holotype deposited under GenBank accession number: OQ683554.

Etymology. From “acus” Latin for needle or pin, referring to the elongated T7.

#### 3.3.2. *Encarsia aisha* Polaszek and Hernández-Suárez **sp. n.**

(Figure 5A–F)

Female. Colour: Antennae brown with F1 and F2 paler. Head dark brown, paler along the sutures. Mesosoma uniformly dark brown. Legs yellow with mid and hind femora, coxae and anterior third of mid tibiae brown, all tarsi pale. Wings hyaline, slightly infuscated below marginal vein; submarginal and marginal veins darker in contrast with the paler stigmal vein.

**Figure 5:**
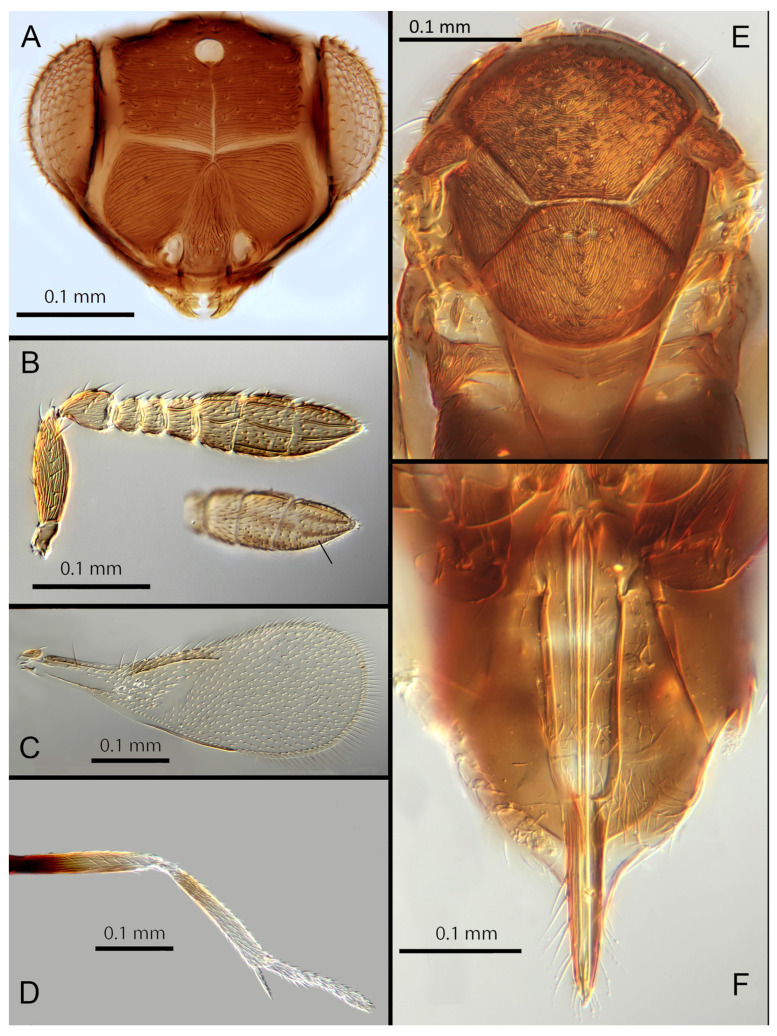
***Encarsia aisha***: (**A**) head; (**B**) antenna, arrow: claval sensorial area; (**C**) fore wing; (**D**) mid leg; (**E**) dorsal mesosoma; (**F**) ovipositor.

Morphology: Head (Figure 5A) with mediofrontal line complete; transfacial line evident; facial lines very broad along their entire lengths. Scrobes with longitudinally aciculate sculpture. Antennal formula (Figure 5B): 1, 1, 3, 3; scape 2.3x pedicel length; pedicel 2x F1; F1 1.2x F2; F2 0.75x F3; funicle 0.45x clava; F6 oblique, claval sensorial area present. Flagellum with the following number of longitudinal sensilla: F1: 0; F2: 0; F3: 1; F4: 2–3; F5: 4–5; F6: 3. Mandibles (Figure 5A) with one large ventral tooth and a bidentate upper tooth. Maxillary palps two-segmented. Mid-lobe of mesoscutum (Figure 5E) with about 34–40 setae; each lateral lobe with two setae; each axilla with one seta; scutellum with four setae and two apparent vestigial setal bases. Sculpture of mesoscutum aciculate; sculpture of axillae and scutellum longitudinal. Fore wing (Figure 5C) with two large setae and four smaller setae on submarginal vein, four setae in basal cell, 6–7 setae on anterior margin of marginal vein, and one seta at the junction of the submarginal vein and parastigma. Linea calva present. Submarginal 0.85x marginal vein. Maximum length of fore wing 2.47x fore wing width, maximum width of wing 5.72x longest seta on marginal fringe. Ovipositor (Figure 5F) 1.47x mid tibial length; third valvulae 0.42x ovipositor length; second valvifer 1.5x third valvula. Mid tibial spur (Figure 5D) 1.07x corresponding basitarsus. Metasomal terga T1–T7 with 0, 2 + 2, 2 + 2, 2 + 2, 1 + 2 + 1, 1 + 2 + 1 and four setae, respectively. T7 (Figure 5F) is extended, as long as ovipositor.

Distribution: COSTA RICA: Alajuela, Heredia.

Material examined: Holotype ♀ COSTA RICA, Alajuela, Est. Biol. Caribe, R. Rincon Forestal 10°53′ N 85°18′ W 400 m 19–20.ii.2003 (J.S. Noyes) [DNA 164: OQ683562] (NHMUK). 1♀ COSTA RICA, Heredia, Estacion Biologica La Selva, 10°26′ N 84°01′ W. 75 m 27–28.ii.2003 (J.S. Noyes) [DNA 146: OQ683562] (ZMUCR).

Remarks: *Encarsia aisha* is morphologically very similar to *E. marynoyesae* in many respects (though distant to it based on DNA). The species can be distinguished from *E. marynoyesae* by the second valvifers almost 2x (1.8) the third valvulae; while they are 1.5x as long in *E. aisha*. In *E. marynoyesaei* the clava is well over 2x the length of the funicle; in *E. aisha* it is less than 2x as long. DNA sequence from holotype and paratype (pooled extraction) deposited under GenBank accession number: OQ683562.

Etymology: Named for Aisha, daughter of the second author (EHS), and sister to Noora; see *E. noora*, below.

#### 3.3.3. *Encarsia aphania* (Polaszek) 1999 (in Martin and Polaszek, 1999: 1556). **comb. nov.**

(Figure 6A–F)

Female. Colour: Antennae pale brown with scape very dark. Head dark brown with pale lines bordering the eyes and extending along the genae towards clypeus, antennal scrobes, a line from the apex of the scrobes to the median ocellus, and a transverse line midway between the antennal scrobes. Mesosoma and metasoma uniformly dark brown. Legs yellow except all coxae and mid and hind femora which are brown. Fore wings faintly infuscate along the submarginal and the marginal veins; submarginal and marginal veins darker in contrast to the paler stigmal vein.

**Figure 6:**
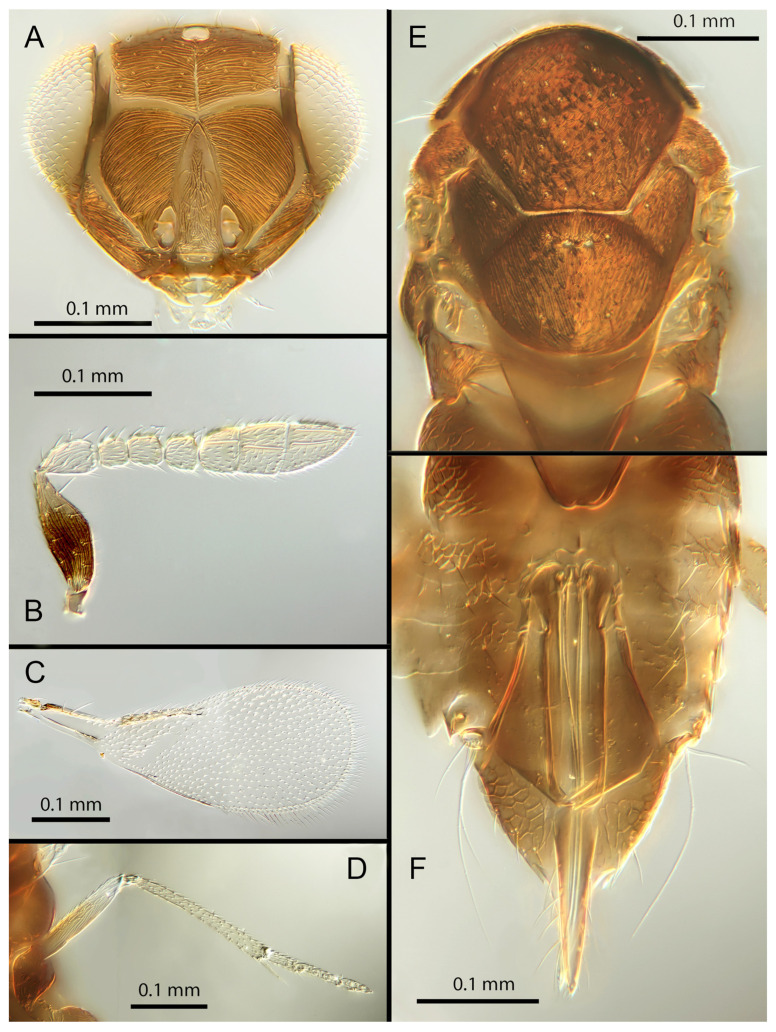
***Encarsia aphania***: (**A**) head; (**B**) antenna; (**C**) fore wing; (**D**) mid leg; (**E**) dorsal mesosoma; (**F**) ovipositor.

Morphology: Head (Figure 6A) with mediofrontal line complete, though fading towards anterior ocellus; transfacial and facial lines very broad along their entire lengths. Scrobes with longitudinally aciculate sculpture centrally, smooth laterally. Antennal formula (Figure 6B): 1, 1, 3, 3; scape 2.39x pedicel; pedicel 2x F1; F1 0.8x F2; F2 equal to F3; funicle 0.67x clava; F4 and F5 partly fused; F6 broadly oblique; claval sensorial area present, indistinct. Flagellum with the following number of longitudinal sensilla: F1: 0; F2: 1; F3: 1; F4: 2–3; F5: 4; F6: 4. Mandibles (Figure 6A) with one large ventral tooth and a broad upper truncation; Maxillary palps two-segmented. Mid-lobe of mesoscutum (Figure 6E) with 30–40 setae; each lateral lobe with one seta; each axilla with one seta; scutellum with four setae. Sculpture of mesoscutum aciculate; sculpture of axillae and scutellum longitudinal. Fore wing (Figure 6C) with two large setae and 4–5 smaller setae on submarginal vein, 4–5 setae in basal cell, 5–7 setae on anterior margin of marginal vein, and 2–3 setae at the distal part of the base. Linea calva present. Submarginal 0.9x marginal vein. Maximum length of fore wing 2.6x fore wing width; maximum width of fore wing 5.2x longest setae on marginal fringe. Ovipositor (Figure 6F) 1.6x mid tibial length; third valvula 0.4x ovipositor length; second valvifer 1.3x third valvula. Mid tibial spur (Figure 6D) as long as corresponding basitarsus. Metasomal terga T1–T7 with 0, 2 + 2, 2 + 2, 1 + 1, 1 + 2 + 1, 1 + 2 + 1 and 5–6 setae, respectively. T7 (Figure 6F) with a pointed, extended apex covering ovipositor.

Distribution: BELIZE: Cayo District; COSTA RICA: Puntarenas.

Hosts: Aleurodicinae: *Aleurodicus pulvinatus* (Maskell), *Azuraleurodicus pentarthrus* Martin; *Nealeurodicus altissimus* (Hempel).

Material examined: Holotype ♀ BELIZE, Cayo District, Chiquibul Forest Reserve, Las Cuevas-Monkey Tail trail, 5.iii.1996 (J.H. Martin #6747) ex *Azuraleurodicus pentarthus* (NHMUK). 13♀ BELIZE, Cayo Las Cuevas, monkey tail trail, 5.iii.1996 (J.H. Martin #6747) ex *Azuraleurodicus pentarthus* [s27, s22, DNA218: OQ683546]. 1♀ BELIZE, Cayo Chiquibul Fr., Monkey tail trail, 21.iii.2003 (J.H. Martin #7768) ex *Nealeurodicus altissimus* on *Inga* sp. (all NHMUK). 1♀ COSTA RICA, Puntarenas, Est. Altamira send. Los Gigantes, 9.vii.2001 (D. Rubi #63984) [DNA213: OQ683545] 1.460 m, LS 331800 572100 (MZUCR)

Remarks: *Encarsia aphania* presents a unique combination of characters and appears to have no very close relatives. Morphologically it is closest to *E. larensis* but is easily distinguishable by the much longer third valvulae relative to the second valvifers (compare Figure 5F and Figure 15F). DNA sequences were obtained from two specimens from Belize (type locality) and Costa Rica, Puntarenas; deposited under GenBank accession numbers: OQ683546, OQ683545.

#### 3.3.4. *Encarsia avida* Polaszek and Hernández-Suárez **sp. n.**

(Figure 7A–F)

Female. Colour: Antennae pale brown, darker on F5–F6 and the scape, pedicel, and radicle. Head brown, paler along the sutures. Mesosoma dark brown with posterior three-quarters of scutellum pale. Metasoma uniformly dark brown. Legs yellow with all coxae, femora, and anterior half of fore leg tibiae brown; all tarsi pale. Wings infuscate below marginal vein; submarginal and marginal veins darker in contrast with the stigmal vein paler.

**Figure 7:**
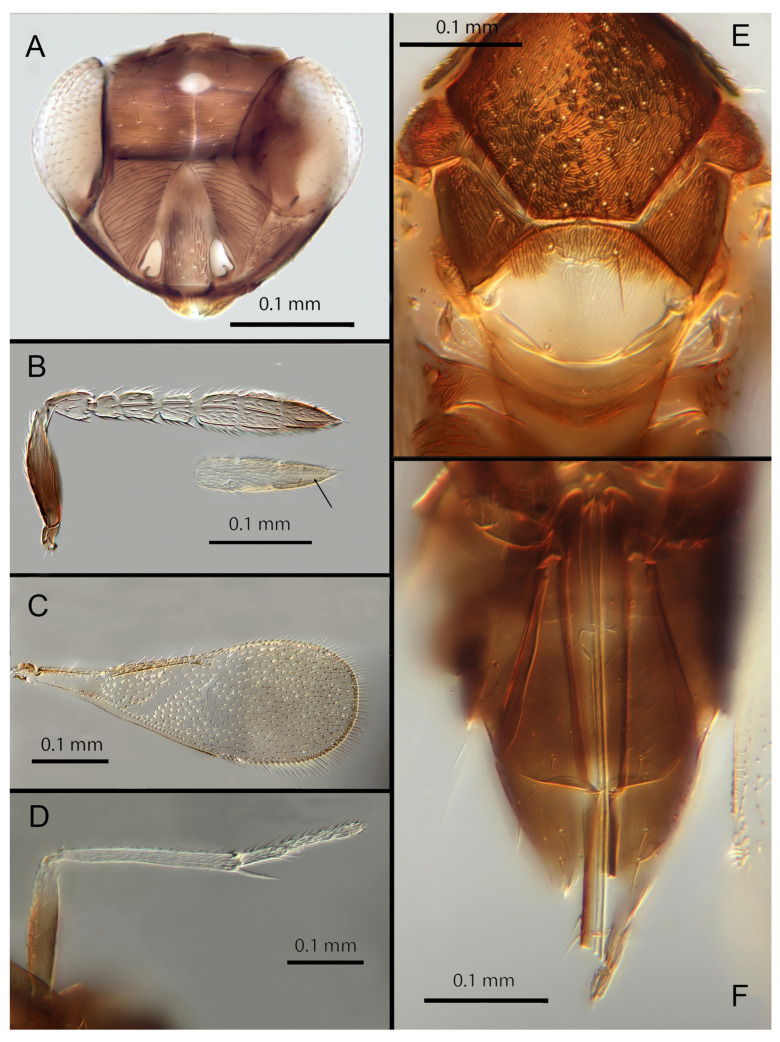
***Encarsia avida***: (**A**) head; (**B**) antenna, arrow: claval sensory area; (**C**) fore wing; (**D**) midleg; (**E**) dorsal mesosoma; (**F**) ovipositor.

Morphology: Head (Figure 7A) with mediofrontal line complete; transfacial line obscure; facial lines present, narrow. Scrobes with longitudinally aciculate sculpture centrally, smooth laterally. Antenna formula (Figure 7B): 1, 1, 3, 3; scape 2.5x pedicel length; pedicel 1.95x F1; F1 0.66x F2; F2 equal to F3; funicle 0.6x clava; F6 perpendicular. Claval sensorial area present, distinct. Flagellum with the following number of longitudinal sensilla: F1: 0; F2: 1–2; F3: 1; F4: 2; F5: 3; F6: 3. Mandibles (Figure 7A) with two small ventral teeth and a truncation. Maxillary palps two-segmented. Mid-lobe of mesoscutum (Figure 7E) with 40–50 setae; each lateral lobe with two setae; each axilla with one seta; scutellum with four setae. Sculpture of mesoscutum aciculate; sculpture of axillae and scutellum longitudinal. Fore wing (Figure 7C) with two large setae and four smaller setae on submarginal vein, four setae in basal cell, eight setae on anterior margin of marginal vein, and one seta at the junction of the submarginal vein and parastigma. Linea calva present. Submarginal 0.67x marginal vein. Maximum length of fore wing 2.8x fore wing width; maximum width of wing 4.7x longest setae on marginal fringe. Ovipositor (Figure 7F) 1.6x mid tibial length; third valvulae 0.4x ovipositor length; second valvifer 1.5x third valvula. Mid tibial spur (Figure 7D) 0.8x corresponding basitarsus. Metasomal terga T1–T7 with 0, 2 + 2, 2 + 2, 2 + 2, 1 + 2 + 1, 1 + 2 + 1 and 4 setae, respectively. T7 (Figure 7F) extended and covering ovipositor (damaged in holotype).

Distribution: COSTA RICA: Heredia.

Material examined: Holotype ♀ COSTA RICA, Heredia, Est. Biol. La Selva, 10°26′ N 84°01′ W 75 m 27–28.ii.2003 (J.S. Noyes) [DNA143: OQ683547] (NHMUK). 

Remarks: *Encarsia avida* appears morphologically close to *E. acusa* with which it shares the color pattern (mesoscutellum anteriorly dark) and wing and antennal morphology. 

The ovipositor in *E. acusa* is longer (1.8x mid tibia; 1.6x in *E. avida*). The most easily appreciated difference is in the sculpture of the frons: *E. avida* has scattered, shallow horizontal grooves (Figure 7A) while *E. acusa* has very dense horizontal grooves (Figure 4A). A similar difference in sculpture is evident on the lateral face. The two species are well-separated based on DNA (Figure 1) with *E. avida* coming out as sister to *E. aphania* with high support (95%). DNA sequence from holotype deposited under GenBank accession number: OQ683547.

Etymology: From “avida -us” meaning “greedy” (Latin).

#### 3.3.5. *Encarsia catula* Polaszek and Hernández-Suárez **sp. n.**

(Figure 8A–F)

Female. Colour: Antennae brown. Head dark brown. Mesosoma uniformly dark brown. Legs yellow with mid and hind femora, coxae, and anterior half of tibiae brown, fore leg femora, coxae, and tibiae dark, all tarsi pale. Wings infuscate below marginal vein, submarginal and marginal veins dark, stigmal vein paler.

**Figure 8:**
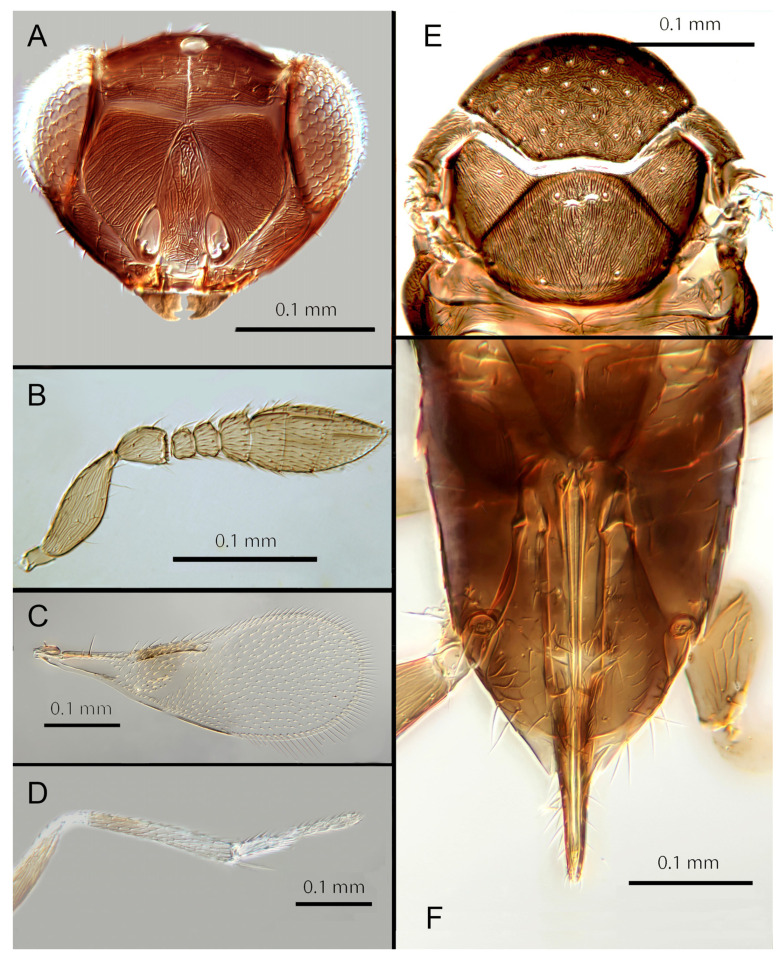
***Encarsia catula***: (**A**) head; (**B**) antenna; (**C**) fore wing; (**D**) midleg; (**E**) dorsal mesosoma; (**F**) ovipositor.

Morphology: Head (Figure 8A) with mediofrontal line complete; transfacial line broad; facial lines present, narrow. Scrobes with longitudinally aciculate sculpture centrally, irregularly aciculate basally. Antenna formula (Figure 8B): 1, 1, 3, 3; scape expanded, 3.1x pedicel length; pedicel 2x F1; F1 0.9x F2; F2 0.8x F3; funicle 0.48x clava; F6 oblique, claval sensorial area present. Flagellum with the following number of longitudinal sensilla: F1: 0; F2: 0; F3: 1; F4: 3; F5: 3; F6: 3. Mandibles (Figure 8A) with one large ventral tooth and a broad truncation. Maxillary palps two-segmented. Mid-lobe of mesoscutum (Figure 8E) with fewer than 30 setae; each lateral lobe with two setae; each axilla with one seta; scutellum with four setae. Sculpture of mesoscutum aciculate; sculpture of axillae and scutellum longitudinal. Fore wing (Figure 8C) with two large setae and four smaller setae on submarginal vein, four setae in basal cell, six setae on anterior margin of marginal vein, and one seta at the junction of the submarginal vein and parastigma. Linea calva present. Submarginal equal to marginal vein. Maximum length of fore wing 2.38x fore wing width, maximum width of wing 4.64x longest setae on marginal fringe. Ovipositor (Figure 8F) 1.48x mid tibial length; third valvulae 0.46x ovipositor length; third valvulae 0.46x ovipositor length; second valvifer 1.2x third valvula. Mid tibial spur (Figure 8D) 1.1x corresponding basitarsus. Metasomal terga T1–T7 with 0, 2 + 2, 2 + 2, 2 + 2, 1 + 2 + 1, 1 + 2 + 1 and four setae, respectively. T7 (Figure 8F) extended although apparently not covering ovipositor.

Distribution: COSTA RICA: Limon.

Material examined: Holotype ♀ COSTA RICA, Limon, Hitoy-Cerere 90°40′ N 83°02′ W, 21–22.iii.2006 (J.S. Noyes) [DNA 278: OQ683547] (NHMUK). 

Remarks: *Encarsia catula* shares aspects of its morphology with *E. marynoyesae*, but can be distinguished by having fewer than 30 setae on the mesoscutum, and third valvulae more than ½ the length of second valvifers (less than ½ as long in *E. marynoyesae*). The two species are relatively close based on DNA (Figure 1). DNA sequence from the holotype is deposited under GenBank accession number: OQ683547.

Etymology: From “catula” meaning dog/whelp (Latin).

#### 3.3.6. *Encarsia cylindrica* Polaszek and Hernández-Suárez **sp. n.**

(Figure 9A–F)

Female. Colour: Antennae pale brown, slightly darker on F6, F1 and the base of the scape, pedicel, and radicle. Head dark brown. Mesosoma uniformly dark brown. Legs yellow with femora and coxae brown, fore legs with dark tibiae, all tarsi pale. Wings infuscate below submarginal vein, marginal and stigmal veins pale. 

**Figure 9:**
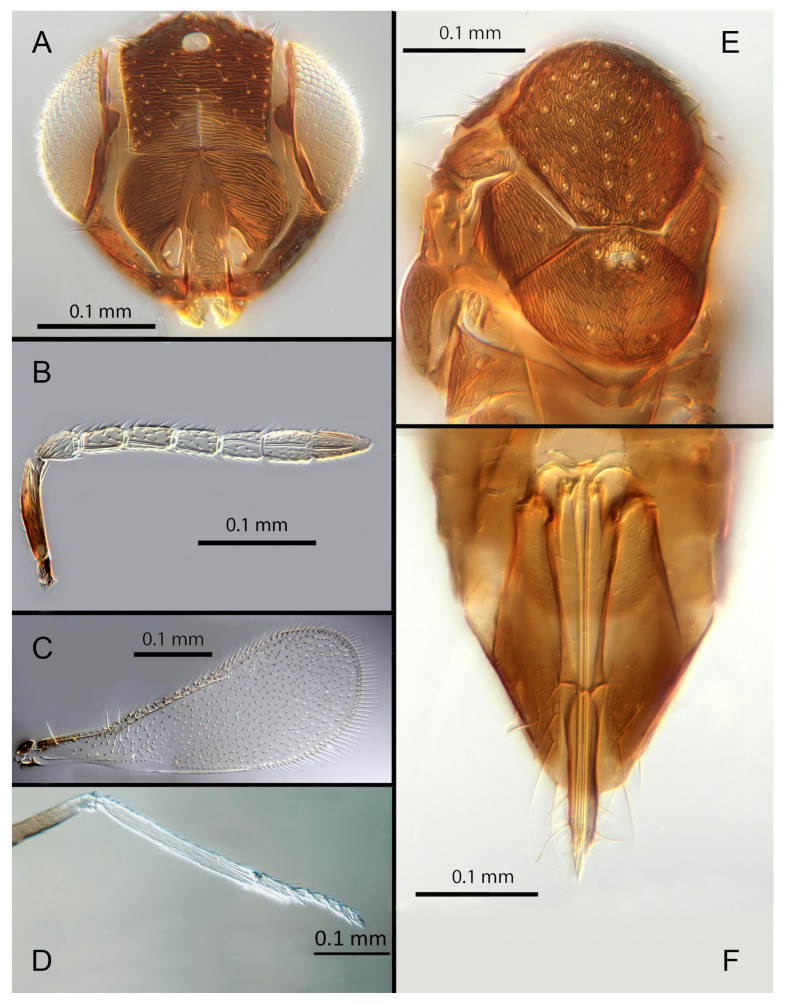
***Encarsia cylindrica***: (**A**) head; (**B**) antenna; (**C**) fore wing; (**D**) midleg; (**E**) dorsal mesosoma; (**F**) ovipositor.

Morphology: Head (Figure 9A) with mediofrontal line incomplete, extending halfway to anterior ocellus; transfacial line obscure; facial lines very broad along their entire lengths. Scrobes with faint longitudinal sculpture apically, irregular/lateral sculpture basally. Antennal formula (Figure 9B): 1, 1, 3, 3; scape 2.75x pedicel length; pedicel equal to F1; F1 0.85x F2; F2 equal to F3; funicle 0.86x clava; F6 perpendicular. Flagellum with the following number of longitudinal sensilla: F1: 2; F2: 2; F3: 2; F4: 2; F5: 3; F6: 3. Mandibles (Figure 9A) with two small teeth and a broad truncation. Maxillary palps two-segmented. Mid-lobe of mesoscutum (Figure 9E) with about 40–50 setae; each lateral lobe with two setae; each axilla with one seta; scutellum with four setae. Sculpture of mesoscutum aciculate; sculpture of axillae and scutellum longitudinal. Fore wing (Figure 9C) with two large setae on submarginal vein and 11 smaller setae, six setae in basal cell, 11 setae on anterior margin of marginal vein, and one seta at the junction of the submarginal vein and parastigma. Linea calva absent. Submarginal 0.62x times marginal vein. Maximum length of fore wing 2.7x fore wing width, maximum width of wing 5.87x longest setae on marginal fringe. Ovipositor (Figure 9F) equal to mid tibial length; third valvula 0.45x ovipositor length; second valvifer 1.3x third valvula. Mid tibial spur (Figure 9D) 1.1x corresponding basitarsus. Metasomal terga T1–T7 with 0, 1 + 1, 1 + 1, 1 + 1, 1 + 2 + 1, 1 + 2 + 1 and six setae, respectively. T7 (Figure 9F) rounded, not extended but covering ovipositor.

Distribution: BRAZIL: Minas Gerais; COSTA RICA: Puntareñas, San Juan; JAMAICA.

Host: Aleurodicinae: *Aleurodicus jamaicensis* Cockerell.

Material examined: Holotype ♀ COSTA RICA, San Juan, Ciudad Colon, Heredia El Rodeo coll parataxonomist 16.ii.1991 [DNA 211] (NHMUK). Paratype 1♀ COSTA RICA, Puntareñas R.F. Golfo Dulce, 24 km W. Piedras Blancas [DNA 209] (MZUCR). 4♀ JAMAICA, Fair Prospect, xii.1968 (K. Heinze) ex *Aleurodicus jamaicensis* [s10] (on 1 slide, USNM). 8♀ BRAZIL, Vicosa, Minas Gerais, 6.xi.1935 (E.J. Hambleton) ex *Aleurothrixus floccosus* (?) (on one slide, one head missing, USNM).

Remarks: *Encarsia cylindrica* appears to be most closely related to *E. erwini*, with which it shares the elongate antenna and lack of a linea calva. It differs from *E. erwini* in having many more setae on the mesoscutum.

Etymology. “cylindrica” refers to the almost uniformly elongate antenna.

#### 3.3.7. *Encarsia diablejo* (Polaszek and Hayat) **comb. n.**

(Figure 10A–F)

*Dirphys diablejo* Polaszek and Hayat, 1992: 189

Female. Unknown. This species is known only from the holotype.

Male. Colour: Antennae uniformly light brown, slightly darker on the base of the scape, pedicel, and radicle. Head brown with paler areas bordering the eyes and extending along the genae towards the clypeus. Mesosoma and metasoma uniformly brown. Legs light brown, the mid and hind tibia pale in contrast to the dark femora; all tarsi pale. Fore wings hyaline, stigmal vein pale in contrast with a darker marginal vein. 

**Figure 10:**
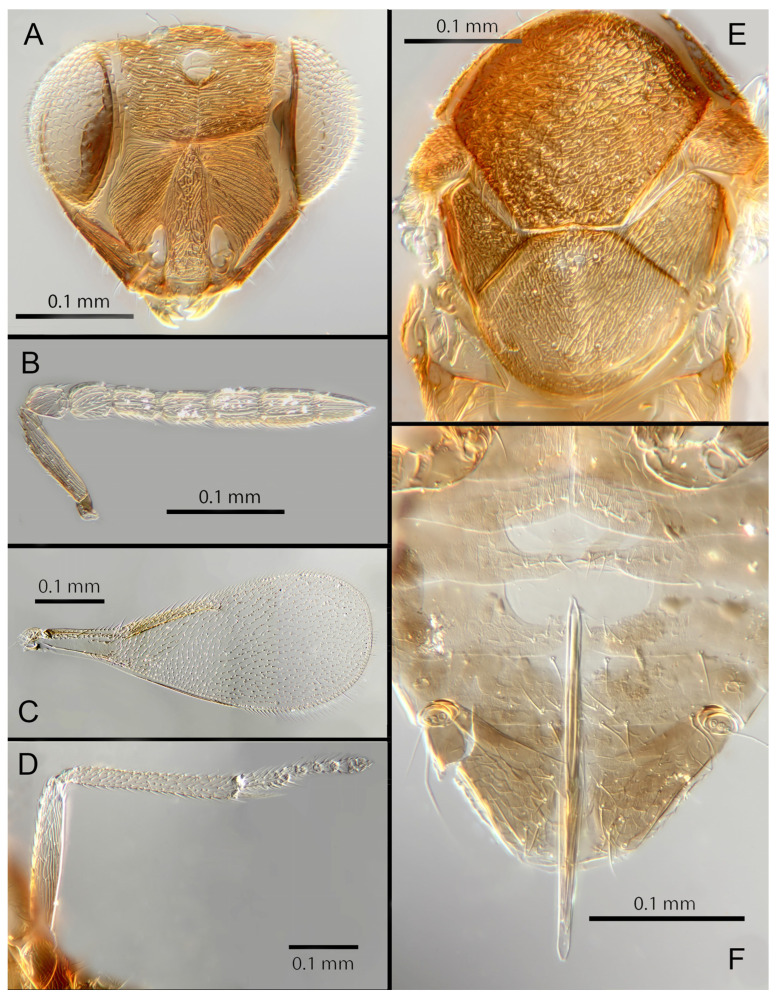
***Encarsia diablejo***: (**A**) head; (**B**) antenna; (**C**) fore wing; (**D**) midleg; (**E**) dorsal mesosoma; (**F**) male genitalia.

Morphology:. Head (Figure 10A) with mediofrontal line complete, though fading towards anterior ocellus; transfacial line complete, narrow; facial lines very broad along their entire lengths. Scrobes entirely with irregular aciculate sculpture. Antennal formula (Figure 10B): 1, 1, 3, 3; scape 2.94x pedicel, F1 subequal to pedicel, F1 0.88X F2, F2 and F3 subequal; funicle 0.89x clava length. Flagellum with the following number of longitudinal sensilla: F1: 7; F2: 6; F3: 7; F4: 8; F5: 9; F6: 7. Mandibles (Figure 10A) with two large pointed teeth and a truncation; maxillary palps two-segmented. Mid-lobe of mesoscutum (Figure 10E) with more than 60 setae; each lateral lobe with one seta; each axilla with one seta; scutellum with four setae and two vestigial setal bases. Sculpture of mesoscutum transverse. Fore wing (Figure 10C) with two large setae and four smaller setae on submarginal vein, five setae in basal cell, 11 setae on anterior margin of marginal vein. Linea calva absent. Submarginal 0.79x marginal vein. Maximum length of fore wing 2.48x fore wing width, maximum width of fore wing 7x longest setae on marginal fringe. Mid tibial spur (Figure 10D) as long as corresponding basitarsus. 

Distribution: PERU: Loreto.

Host: Unknown.

Material examined: Holotype ♂ PERU, Loreto, Iquitos, Granja Unap, 9.ii.1984 (L. Huggert #BM 1984-337) [s26] (NHMUK).

Remarks: For the purposes of the identification key, we have assumed that the (unknown) female of *E. diablejo* shares the wing and mesosomal sculpture characters with the male; the combination of which is unique in the *Encarsia mexicana* species-group.

#### 3.3.8. *Encarsia dichaeta* Polaszek and Hernández-Suárez **sp. n.**

(Figure 11A–F)

Female. Colour: Antennae light brown, slightly darker on the base of the scape, pedicel, and radicle. Head dark brown with pale lines bordering the eyes and extending along the genae towards clypeus. Mesosoma and metasoma uniformly dark brown. Legs yellow with femora and coxa brown, tibia, and all tarsi pale. Wings infuscate below submarginal and marginal veins, stigmal vein pale.

**Figure 11:**
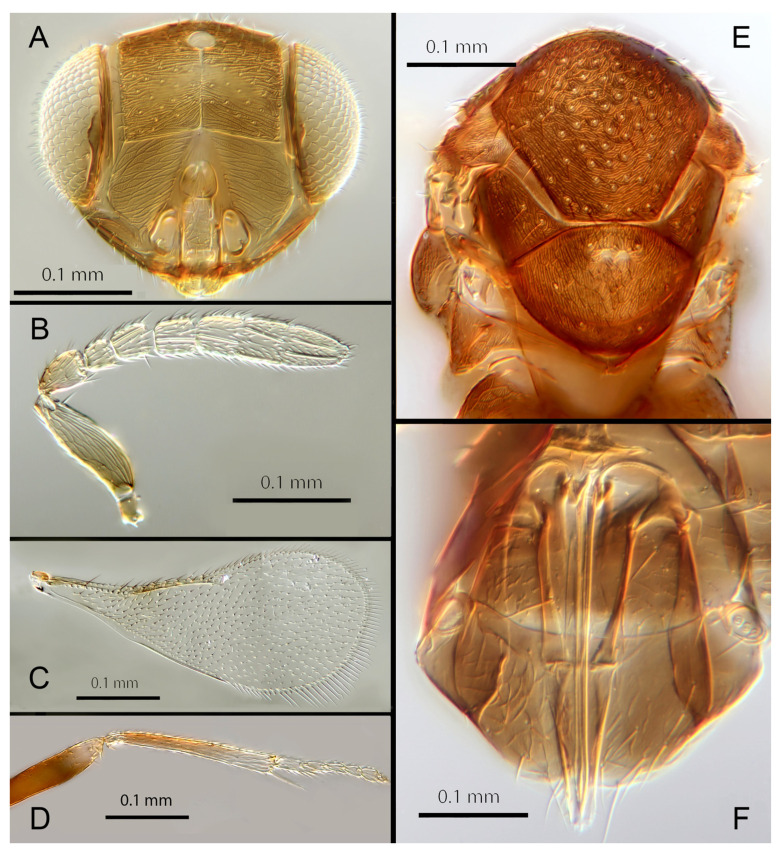
***Encarsia dichaeta***: (**A**) head; (**B**) antenna; (**C**) fore wing; (**D**) midleg; (**E**) dorsal mesosoma; (**F**) ovipositor.

Morphology: Head (Figure 11A) with mediofrontal line complete; transfacial line narrow; facial lines relatively narrow along their entire lengths. Scrobes with irregular aciculate sculpture. Antennal formula (Figure 11B): antennal formula: 1, 1, 3, 3; scape 2.59x pedicel length; pedicel 1.85x F1; F1 0.85x F2; F2 0.8x F3; funicle 0.65x clava length; F6 perpendicular. Flagellum with the following number of longitudinal sensilla: F1:0; F2:1; F3:1; F4:1–2; F5:1–2; F6:1–2. Mandibles (Figure 11A) with two small teeth and a truncation. Maxillary palps two-segmented. Mid-lobe of mesoscutum (Figure 11E) with more than 50 setae; each lateral lobe with two setae; each axilla with two setae scutellum with four setae andtwo vestigial setal bases. Sculpture of mesoscutum aciculate; sculpture of axillae and scutellum longitudinal. Fore wing (Figure 11C) with two large setae and 7–8 smaller setae on submarginal vein, 14 setae in basal cell, eight setae on anterior margin of marginal vein, and one seta at the junction of the submarginal vein and parastigma. Linea calva absent. Submarginal 0.85x times marginal vein. Maximum length of fore wing 2.48x fore wing width, maximum width of wing 5.45x longest setae on marginal fringe. Ovipositor (Figure 11F) 0.93x mid tibial length; third valvulae 0.45x ovipositor; second valvifer 1.6x third valvula. Mid tibial spur (Figure 11D) equal to corresponding basitarsus. Metasomal terga T1–T7 with 0, 1 + 1, 1 + 1, 1 + 1, 1 + 2 + 1, 1 + 2 + 1 and 12 setae, respectively. T7 (Figure 11F) rounded, not extended but covering ovipositor.

Distribution: BRAZIL: Bahia; COSTA RICA: Guanacaste, Alajuela, Heredia; ECUADOR: Napo River.

Host: Aleurodicinae: *Aleurodicus flavus* Hempel, *Aleurodicus* sp.

Material examined: Holotype 1♀ COSTA RICA, Alajuela, P.N. Arenal Sendero Pilon, 10°27′ N 84°45′ W 600 m 25.ii.2003 (J.S. Noyes) [DNA 136: OQ683552] (NHMUK). Paratypes 3♀ COSTA RICA, Alajuela, P.N. Arenal, Sendero Pilon, 10°27′ N 84°45′ W 600 m 25.ii.2003 (J.S. Noyes), [DNA 132: OQ683550, 133: OQ683552, 135: OQ683550] (2♀ NHMUK, 1♀ MZUCR). 1♀ COSTA RICA, Heredia, Est. Biol. La Selva, 10°26′ N 84°01′ W 75 m 27–28.ii.2003 (J.S. Noyes) [DNA151: OQ683549] (UCRC); 1♀ COSTA RICA, P.N. Guanacaste, Est. Pitilla (ACG), 11°00′ N. 85°26′ W. 700 m MT/YPT (J.S. Noyes) [DNA 208: OQ683551] (NHMUK). 27♀♀ BRAZIL, Bahia (Gregorio Bondar # nº65b) ex *Aleurodicus flavus* (on 5 slides; USNM). 5♀ ECUADOR, Napo, Camino Añangucocha, 29.iii.04 (H. Evans) ex *Aleurodicus* sp. (NHMUK).

Remarks: There are some colour differences between the Costa Rican specimens and those from Brazil, the latter having the metasoma distally paler. Further studies on fresh material, in particular DNA sequencing, will be needed to confirm their status. DNA sequences were obtained from the holotype and five paratypes, deposited under GenBank accession numbers: OQ683550, OQ683552, OQ683551 and OQ683549 (three paratype specimens were pooled for extraction).

Etymology. “*dichaeta*” refers to the two setae on each axilla, unique for the genus.

#### 3.3.9. *Encarsia encantadora* (Polaszek and Hayat) **comb. n.**

(Figure 12A–F)

*Dirphys encantadora* Polaszek and Hayat, 1992: 191.

Female. Colour: Antennae pale brown/yellow with dark scape and radicle. Head brown with paler areas bordering the eyes and extending along the genae towards the clypeus, antennal scrobes, a line from the apex of the scrobes to the median ocellus, and a transverse line midway between the antennal sockets and the median ocellus. Mesosoma brown in holotype but with posterior three-quarters of scutellum pale in Mexican specimens. Legs light brown, the mid and hind tibiae pale in contrast to the dark femora and coxa, all tarsi pale. Wings hyaline, faintly infuscate below the marginal vein, stigmal vein pale in contrast with a darker marginal vein. 

**Figure 12:**
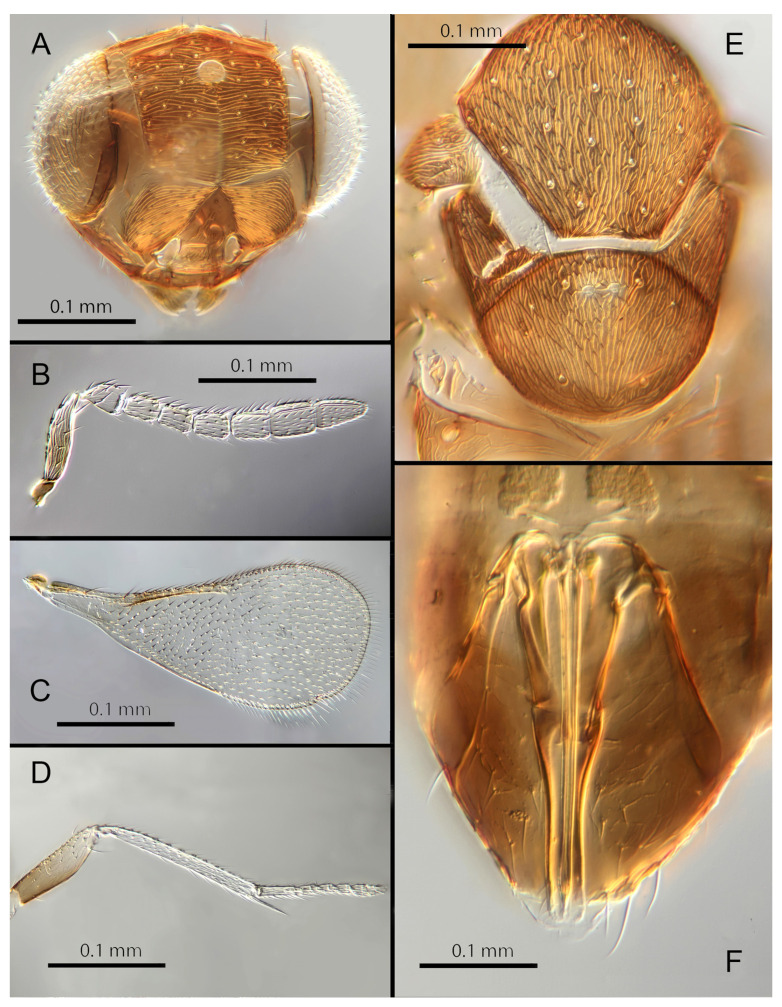
***Encarsia encantadora***: (**A**) head; (**B**) antenna; (**C**) fore wing; (**D**) midleg; (**E**) dorsal mesosoma; (**F**) ovipositor.

Morphology: Head (Figure 12A) with mediofrontal line complete; transfacial line evident; facial lines very broad along their entire lengths. Scrobes with longitudinally aciculate sculpture. Antennal formula (Figure 12B): 1, 1, 3, 3; scape 2.3x pedicel length; pedicel equal to F1; F1 to F3 funicle segments all subequal in length; funicle 0.75x clava length; F6 perpendicular. Flagellum with the following number of longitudinal sensilla: F1: 1–2; F2: 1; F3: 1; F4: 2–3; F5: 3; F6: 4. Mandibles (Figure 12A) with two small ventral teeth and a broad truncation dorsally. Maxillary palps two-segmented. Mid-lobe of mesoscutum (Figure 12E) with fewer than 30 setae; each lateral lobe with three setae; each axilla with one seta; scutellum with four setae. Sculpture of mesoscutum aciculate; sculpture of axillae and scutellum longitudinal. Fore wing (Figure 12C) with two setae on submarginal vein, 7–9 setae in basal cell, 7–11 setae on anterior margin of marginal vein, and one seta at the junction of the submarginal vein and parastigma. Linea calva absent. Submarginal 0.73x times marginal vein. Maximum length of fore wing 2.52x fore wing width, maximum width of fore wing 6.9x longest setae on marginal fringe. Ovipositor (Figure 12F) 0.82x mid tibial length; third valvula 0.55x ovipositor length; second valvifer 0.79x third valvula. Mid tibial spur (Figure 12D) 0.87x corresponding basitarsus. Metasomal terga T1–T7 with 0, 1 + 1, 1 + 1, 1 + 1, 1 + 2 + 1, 1 + 2 + 1 and four setae, respectively. T7 (Figure 12F) rounded and covering ovipositor third valvula.

Distribution: ECUADOR: Napo; MEXICO: Tabasco.

Host: Aleurodicinae: *Nealeurodicus altissimus* (Quaintance)

Material examined: Holotype ♀ ECUADOR, Napo, Sacha, 5.iii.1983 (L. Huggert) (NHMUK). 1♀, fragments of a second ♀: MEXICO, Tabasco, San Francisco del Peal, 1.vii.1897 (C.H.T. Townsend) ex *Nealeurodicus altissimus* (Quaintance) on *Lippia myriophala* Schltdl. and Cham. (USNM; on slide with *E. mexicana* type material).

Remarks: *Encarsia encantadora* is morphologically closest to *E. erwini,* differing from that species mainly in having the third valvulae longer than the second valvifers. The fore wing is also broader in *E. encantadora,* especially measured relative to the longest wing fringe setae (compare Figure 11C and Figure 12C). 

#### 3.3.10. *Encarsia erwini* Polaszek and Hernández-Suárez **sp. n.**

(Figure 13A–F)

Female. Colour: Antennae entirely pale, only the scape and radicle dark. Head dark brown. Mesosoma uniformly dark brown. Legs entirely pale except all coxae brown (female paratype with some infuscation on the hind femora).

**Figure 13:**
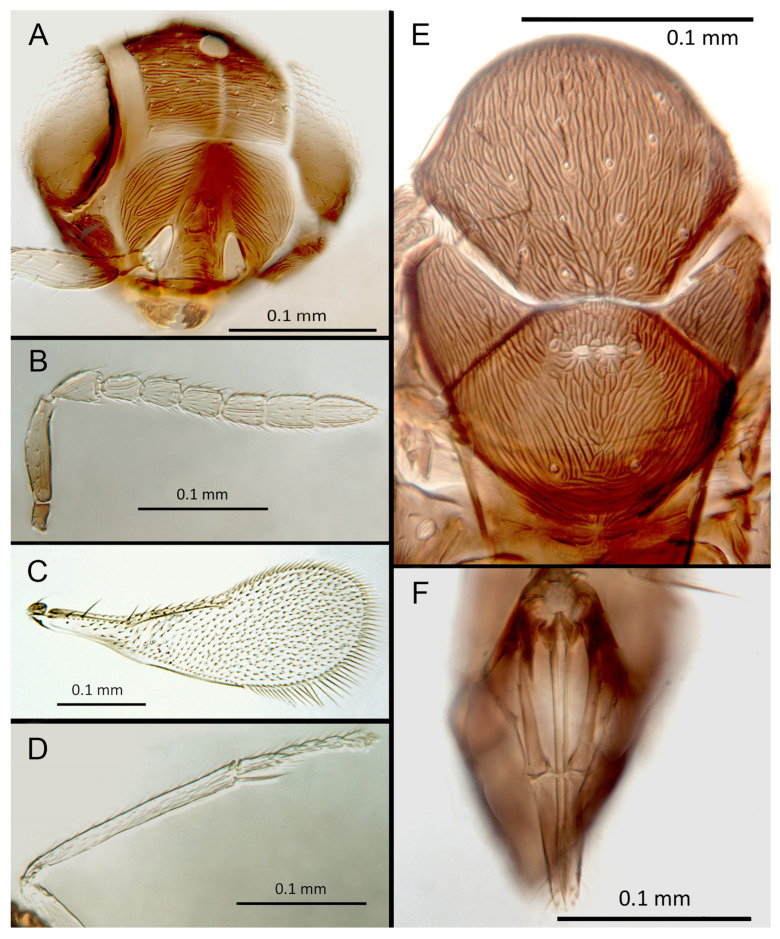
***Encarsia erwini***: (**A**) head; (**B**) antenna; (**C**) fore wing; (**D**) midleg; (**E**) dorsal mesosoma; (**F**) ovipositor.

Morphology: Head (Figure 13A) with mediofrontal line complete; transfacial and facial lines very broad along their entire lengths. Scrobes with irregularly aciculate sculpture centrally. Antennal formula (Figure 13B): 1, 1, 3, 3; scape 2.4x pedicel length; pedicel 1.2x F1 equal to F2; F2 equal to F3; funicle 0.77x clava; F6 perpendicular. Flagellum with the following number of longitudinal sensilla: F1: 1; F2: 1; F3: 1; F4: 1; F5: 2; F6: 2. Mandibles (Figure 13A) with 2 ventral teeth and a truncation dorsally. Maxillary palps two-segmented. Mid-lobe of mesoscutum (Figure 13E) with about 18 setae; each lateral lobe with three setae; each axilla with one seta; scutellum with four setae. Sculpture of mesoscutum and axillae longitudinally aciculate; sculpture of scutellum longitudinal, transverse apically. Fore wing (Figure 13C) with two large setae on submarginal vein and five smaller setae above, six setae in basal cell, seven setae on anterior margin of marginal vein, and one large seta at the junction of the submarginal vein and parastigma. Linea calva absent. Submarginal vein approximately equal in length to marginal vein. Maximum length of fore wing 2.9x fore wing width, maximum width of wing 3.75x longest seta on marginal fringe.

Ovipositor (Figure 13F) equal to mid tibial length; third valvula 0.44x ovipositor length; second valvifer 1.3x third valvula. Mid tibial spur (Figure 13D) 1.0x corresponding basitarsus. Metasomal terga T1–T7 with 0, 1 + 1, 1 + 1, 1 + 1, 1 + 2 + 1, 2 + 2 and seven setae, respectively. T7 (Figure 13F) conical, not extended but just covering ovipositor.

Distribution: ECUADOR: Napo.

Material examined: Holotype ♀ ECUADOR, Napo, transect ent. 1 km S. Onkone Gare Camp, Res. Etnica Waorani 220 m 0°39′10″ S 76°26′00″ W TL Erwin et al. fogging t.f. forest. Lot #1255 8.x.1995 (UCRC; 52715). Paratype ♀, same data as holotype except 4.x.1996 (NHMUK).

Remarks: *Encarsia erwini* appears to be most closely related to *E. cylindrica*, with which it shares the elongate antenna and lack of a linea calva. It differs from *E. cylindrica* in having far fewer setae on the mesoscutum. *E. erwini* is also morphologically close to *E. encantadora*, differing from that species mainly in having the third valvulae shorter than the second valvifers. The fore wing is also broader in *E. encantadora*, especially measured relative to the longest wing fringe setae.

Etymology: Named for the late Terry Erwin (1940–2020), prolific collector of insects, especially in the rain forest canopy of Ecuador.

#### 3.3.11. *Encarsia fredbennetti* Polaszek and Hernández-Suárez **sp. n.**

(Figure 14A–E)

Female. Colour: Antennae uniformly pale brown. Head brown with pale lines bordering the eyes and extending along the genae towards clypeus. Mesosoma dark brown with most of scutellum and post-scutellum pale; metasoma uniformly brown. Legs yellow with dark coxae, femora and anterior third of hind leg tibia. Wings infuscate below marginal vein, stigmal vein pale in contrast with darker marginal vein.

**Figure 14:**
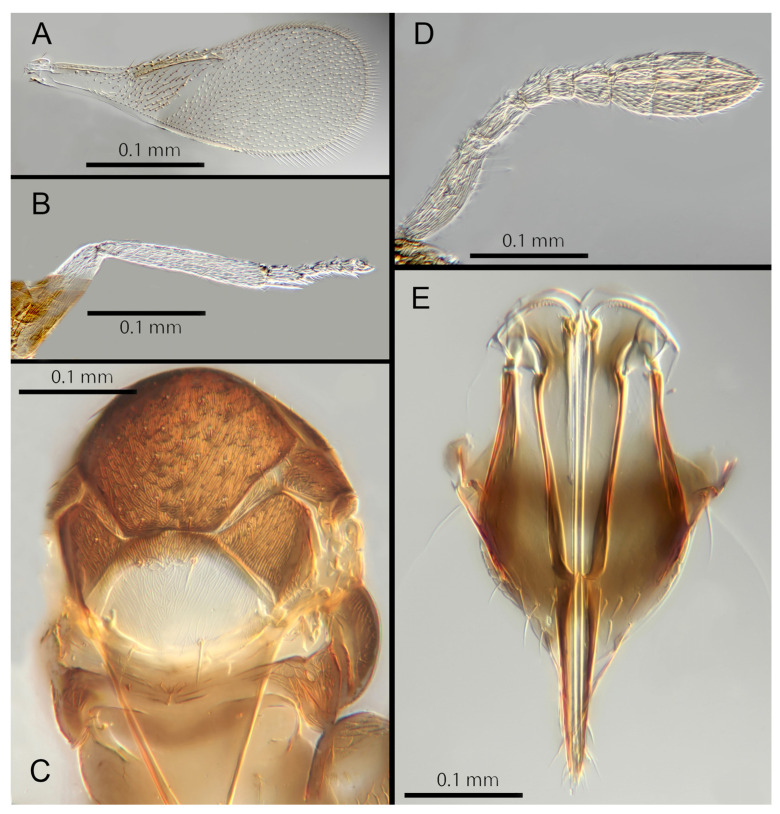
***Encarsia fredbennetti***: (**A**) fore wing; (**B**) mid leg; (**C**) dorsal mesosoma; (**D**) antenna; (**E**) ovipositor.

Morphology: Head (not shown) with all facial lines obscure in holotype (head absent in paratypes). Scrobes with longitudinally aciculate sculpture. Scrobes with longitudinally aciculate sculpture. Antennal formula (Figure 14D): 1, 1, 3, 3 (though could be interpreted as 1, 1, 2, 4); scape 2.39x pedicel length; pedicel 1.9x F1; F1 0.8x F2; F2 0.9x F3; funicle 0.6x clava; F6 perpendicular. Flagellum with the following number of longitudinal sensilla: F1: 0; F2: 1; F3: 1; F4: 2–3; F5: 3; F6: 3. Mandibles with one ventral tooth and a truncation. Maxillary palps two-segmented. Mid-lobe of mesoscutum (Figure 14C) with 30–40 setae; each lateral lobe with two setae; each axilla with one seta; scutellum with four setae. Sculpture of mesoscutum aciculate; sculpture of axillae and scutellum longitudinal. Fore wing (Figure 14A) with two large setae and four smaller setae on submarginal vein, 4–5 setae in basal cell, seven setae on anterior margin of marginal vein. Linea calva present. Submarginal 0.93x marginal vein. Maximum length of fore wing 3.8x fore wing width, maximum width of wing 3.7x longest setae on marginal fringe. Ovipositor (Figure 14E) 1.3x mid tibial length; third valvulae 0.5x ovipositor length; second valvifer equal to third valvula. Mid tibial spur (Figure 14B) equal to corresponding basitarsus. Metasomal terga T1–T7 with 0, 2 + 2, 2 + 2, 2 + 2, 1 + 2 + 1, 1 + 2 + 1 and four setae, respectively. T7 (Figure 14E) extended, covering ovipositor.

Distribution: TRINIDAD: St Augustine.

Host: Aleurodicinae.

Material examined: Holotype ♀ TRINIDAD, [St Augustine] ICTA [Imperial College of Tropical Agriculture] xii.1953 FD Bennett ex whitefly on cocoa (NHMUK); Paratype ♀ TRINIDAD, St Augustine, ex Aleurodicinae [DNA215: OQ683559] (NHMUK); Paratype ♀ TRINIDAD, Mt St Benedict, ex whitefly Coll. M. Jagroep [DNA216] (NHMUK).

Remarks: *Encarsia fredbennetti* is morphologically closest to *E. mexicana* and *E. inbioa* from which it differs by the enlarged clava. It is also molecularly closest to *E. mexicana*. Deposited under GenBank accession number: OQ683559.

Etymology: Named for the late Fred D. Bennett (1925–2021), former Director of the Commonwealth Institute of Biological Control and avid collector of parasitoids during much of his long life. 

#### 3.3.12. *Encarsia inbioa* Polaszek and Hernández-Suárez **sp. n.**

(Figure 15A–F)

Female. Colour: Antennae light brown slightly darker at F5 and F6, the base of the scape, pedicel, and radicle. Head brown with pale lines bordering the eyes and extending along the genae towards clypeus. Mesosoma dark brown but with posterior quarter of scutellum pale; metasoma uniformly dark brown. Legs yellow with dark coxae, femora and anterior third of hind leg tibia. Wings infuscate below marginal vein, stigmal vein pale in contrast with the darker marginal vein.

**Figure 15:**
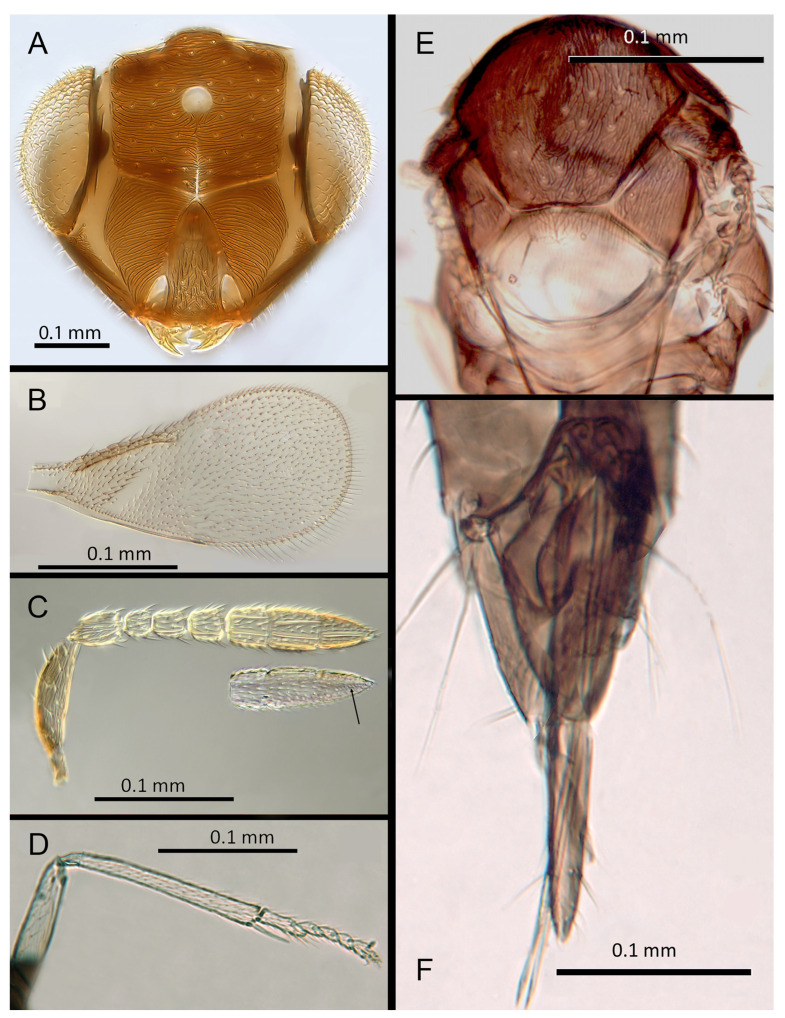
***Encarsia inbioa***: (**A**) head; (**B**) fore wing; (**C**) antenna, arrow: claval sensory area; (**D**) mid leg; (**E**) dorsal mesosoma; (**F**) ovipositor.

Morphology: Head (Figure 15A) with mediofrontal line incomplete, reaching to less than half the distance to the frontal ocellus; transfacial line evident, narrow; facial lines very broad along their entire lengths, particularly at the level of the lower eye. Scrobes with aciculate/reticulate sculpture basally and centrally, smooth apically and apico-laterally. Antennal formula (Figure 15C): 1, 1, 3, 3; scape 2.39x pedicel length; pedicel 1.9x F1; F1 0.8x F2; F2 0.9x F3; funicle 0.6x clava; F6 perpendicular. Flagellum with the following number of longitudinal sensilla: F1: 0; F2: 1; F3: 1; F4: 2–3; F5: 3; F6: 3. Mandibles (Figure 15A) with two large teeth and a truncation. Maxillary palps two-segmented. Mid-lobe of mesoscutum (Figure 15E) with 30–40 setae; each lateral lobe with two setae; each axilla with one seta; scutellum with four setae. Sculpture of mesoscutum aciculate; sculpture of axillae and scutellum longitudinal. Fore wing (Figure 15B) with two large setae and four smaller setae on submarginal vein, 4–5 setae in basal cell, seven setae on anterior margin of marginal vein. Linea calva is present. Submarginal 0.93x marginal vein. Maximum length of fore wing 3.8x fore wing width, maximum width of wing 3.7x longest setae on marginal fringe. Ovipositor (Figure 15F) 1.3x mid tibial length; third valvulae 0.5x ovipositor length; second valvifer equal to third valvula. Mid tibial spur (Figure 15D) equal to corresponding basitarsus. Metasomal terga T1–T7 with 0, 2 + 2, 2 + 2, 2 + 2, 1 + 2 + 1, 1 + 2 + 1 and four setae, respectively. T7 (Figure 15F) extended and covering ovipositor.

Distribution: COSTA RICA: Alajuela.

Material examined: Holotype ♀ COSTA RICA, Alajuela, P.N. Arenal, Sendero Pilon, 26.ii.2003 (J.S. Noyes) [DNA 128: OQ683553] 600 m 10°27′ N 84°43′ W (NHMUK).

Remarks: *Encarsia inbioa* is morphologically closest to *E. fredbennetti* from which it differs by the non-enlarged clava. It is, perhaps surprisingly, molecularly closest to *E. svetlana*. Deposited under GenBank accession number: OQ683553.

Etymology: Named for INBio (Instituto Nacional de Biodiversidad) the national institute for biodiversity and conservation in Costa Rica.

#### 3.3.13. *Encarsia larensis* (Chavez) **comb. n.**

*Dirphys larensis* Chavez, 1996: 11

(Figure 16A–F)

Female. Colour:. Antennae pale brown with slightly darker clava and pedicel. Head dark brown with paler areas bordering the eyes and extending along the genae towards the clypeus. Mesosoma and metasoma uniformly dark brown, third valvulae dark brown contrasting with the rest of ovipositor. Legs pale, mid and hind femur, and coxae brown, anterior third of mid leg tibia brown. Wings infuscate below the submarginal and marginal vein; marginal and stigmal veins dark. 

**Figure 16:**
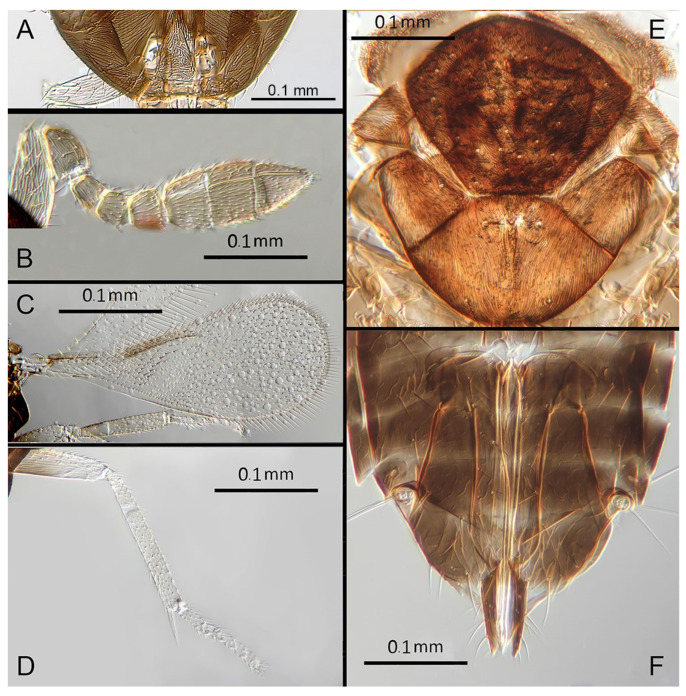
***Encarsia larensis***: (**A**) head; (**B**) antenna; (**C**) fore wing; (**D**) mid leg; (**E**) dorsal mesosoma; (**F**) ovipositor.

Morphology: Head (Figure 16A) with mediofrontal line complete, reaching to the frontal ocellus; other facial lines obscure in paratypes examined due to mounting method. Scrobes with coarse longitudinal aciculate sculpture becoming irregular towards clypeus. Antennal formula (Figure 16B): 1, 1, 3, 3; scape expanded, 2.4–2.5x pedicel length; pedicel 1.9x F1; F1 equal to F2; F2 0.8x F3; funicle 0.58x clava; F6 oblique. 

Flagellum with the following number of longitudinal sensilla: F1: 0; F2: 0; F3: 1; F4: 4; F5: 5–6; F6: 4. Mandibles (not shown) with one large ventral tooth and a bidentate upper tooth. 

Maxillary palps two-segmented. Mid-lobe of mesoscutum (Figure 16E) with 46–60 setae; each lateral lobe with two setae; each axilla with one seta; scutellum with four setae and two vestigial setal bases. Sculpture of mesoscutum aciculate; sculpture of axillae and scutellum longitudinal. Fore wing (Figure 16C) with two large setae and 3–4 smaller setae on submarginal vein, 7–9 setae on anterior margin of marginal vein, and one seta at the junction of the submarginal vein and parastigma. Linea calva present. Submarginal equal to marginal vein; Maximum length of fore wing 2.54x fore wing width; maximum width of fore wing 6.7x longest setae on marginal fringe. Ovipositor (Figure 16F) 1.30x mid tibial length; third valvula 0.28x ovipositor length; second valvifer 2.4x third valvula. Mid tibial spur (Figure 16D) 1.15x corresponding basitarsus. Metasomal terga T1–T7 with 0, 2 + 2, 2 + 2, 2 + 2, 1 + 2 + 1, 1 + 2 + 1 and four setae, respectively. T7 (Figure 16F) rounded not covering ovipositor third valvula.

Male. Colour: Head light brown. Mesosoma and gaster uniformly brown but posterior third of mesoscutum, anterior third of axillae and scutellum yellow. Legs brown. Fore wings hyaline.

Morphology: Similar to that of female, except antennal formula, flagellum with longitudinal sensilla on all segments and funicle segments subequal in length.

Distribution: VENEZUELA: Cabudare, Lara.

Host: Aleurodicinae: *Aleurodicus pulvinatus* (Maskell).

Material examined: 1♀, 1♂: VENEZUELA, Cabudare, Lara, i.1994 (A. Chavez and F. Díaz) ex *Aleurodicus pulvinatus* on *Hura crepitans* L. (NHMUK).

Remarks: *Encarsia larensis* appears morphologically closest to *E. marynoyesae* from which it differs in having a much longer funicle, and shorter ovipositor. No molecular data were available for this species. Chavez (1996) recorded 16 individuals of *E. larensis* within a single whitefly host.

#### 3.3.14. *Encarsia marynoyesae* Polaszek and Hernández-Suárez **sp. n.**

(Figure 17A–F)

Female. Colour: Antennae brown with F1 and F2 paler. Head dark brown with pale lines bordering the eyes and extending along the genae towards clypeus. Mesosoma uniformly dark brown. Legs yellow with mid and hind femora, coxa and anterior third of tibia brown, all tarsi pale. Wings slightly infuscated below anterior half of submarginal vein, stigmal vein pale in contrast with a darker marginal vein. 

**Figure 17:**
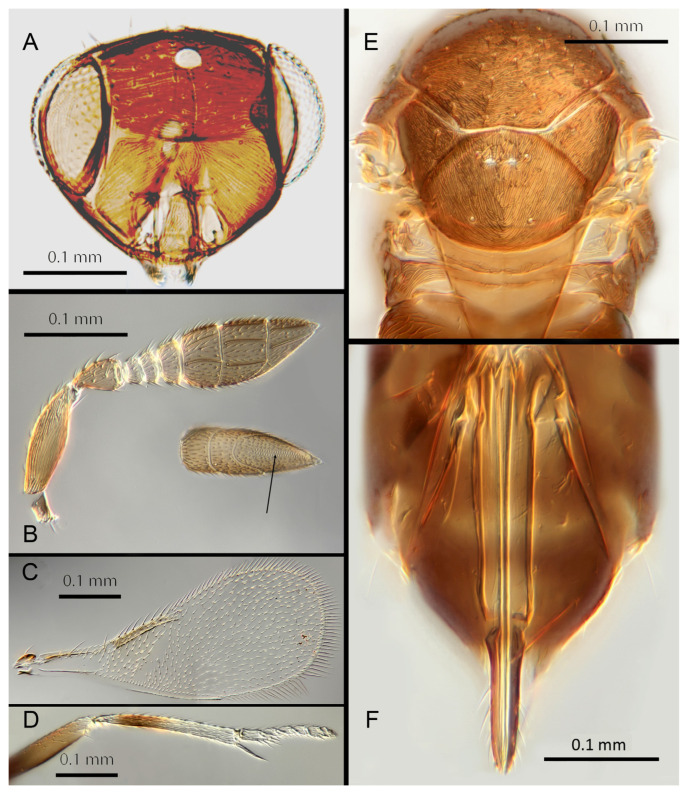
***Encarsia marynoyesae***: (**A**) head; (**B**) antenna, arrow: claval sensory area; (**C**) fore wing; (**D**) mid leg; (**E**) dorsal mesosoma; (**F**) ovipositor.

Morphology: Head (Figure 17A) with mediofrontal line complete; transfacial line narrow; facial lines very broad along their entire lengths. Scrobes with irregular aciculate sculpture. Antennal formula (Figure 17B): 1, 1, 3, 3; scape slightly expanded, 2.53x pedicel length; pedicel 2.85x F1; F1 equal to F2; F2 0.8x F3; funicle 0.36x clava; F5 and F6 strongly oblique, claval sensorial complex developed. Flagellum with the following number of longitudinal sensilla: F1: 0; F2: 0–1; F3: 1; F4: 3; F5: 4; F6: 4. Mandibles (Figure 17A) with one small ventral tooth and a bidentate upper tooth. Maxillary palps two-segmented. Mid-lobe of mesoscutum (Figure 17E) with approximately 40 setae; each lateral lobe with two setae; each axilla with one seta; scutellum with four setae and two vestigial bases. Sculpture of mesoscutum aciculate; sculpture of axillae and scutellum longitudinal. Fore wing (Figure 17C) with two large setae and four smaller setae on submarginal vein, four setae in basal cell, 6–7 setae on anterior margin of marginal vein, and one seta at the junction of the submarginal vein and parastigma. Linea calva present. Submarginal equal to marginal vein. Maximum length of fore wing 2.57x fore wing width, maximum width of wing 6.2x longest setae on marginal fringe. Ovipositor (Figure 17F) 1.4x mid tibial length; third valvulae 0.35x ovipositor length; second valvifer 1.9x third valvula. Mid tibial spur (Figure 17D) 1.07x corresponding basitarsus. Metasomal terga T1–T7 with 0, 1 + 1, 1 + 1, 1 + 1, 1 + 2 + 1, 1 + 2 + 1 and 4 setae, respectively. T7 (Figure 17F) extended although apparently not covering ovipositor.

Distribution: COSTA RICA (Alajuela).

Material examined: Holotype ♀ COSTA RICA, Alajuela, Est. Caribe Reserva Rincón Forestal, 19–20.ii.2003 (J.S. Noyes) [DNA 163: OQ683563] 400 m, 10°53′ N 85°18′ W (NHMUK). Paratype 1♀ COSTA RICA, Alajuela, Est. Caribe Reserva Rincón Forestal, 19–20.ii.2003 (J.S. Noyes) [DNA167] (NHMUK).

Remarks: *Encarsia marynoyesae* is morphologically very similar to *E. aisha* in many respects (though distant to it based on DNA). The species can be distinguished by *E. marynoyesae* having the second valvifers almost 2x (1.8) the third valvulae; while they are 1.5x as long in E. *aisha*. In E. *marynoyesae* the clava is well over 2x the length of the funicle; in E. *aisha* it is less than 2x as long. *E. marynoyesae* also shares aspects of morphology with *E. catula*, but can be distinguished by having more than 30 setae on the mesoscutum, and V3 less than ½ the length of V2 (much more than ½ as long in *E. catula*). Sequence deposited under GenBank accession number: OQ683563.

Etymology. Named for Mary Noyes MBE (Member of the Order of the British Empire). 

#### 3.3.15. *Encarsia mendesi* (Polaszek and Hayat) **comb. n.**

(Figure 18A–F)

*Dirphys mendesi*, Polaszek and Hayat 1992: 191

Female. Colour: Antennae brown, paler on their ventral halves. Head dark brown with pale lines bordering the eyes and extending along the genae towards clypeus, antennal scrobes, a line from the apex of the scrobes to the median ocellus, and a transverse line midway between the antennal sockets and the median ocellus centrally bordering the dorsal end of antennal scrobes. Mesosoma and metasoma uniformly dark brown. Legs pale yellow, with coxae and hind femora dark brown. Wings infuscated below the submarginal and marginal vein; stigmal vein pale in contrast with a darker marginal vein. 

**Figure 18:**
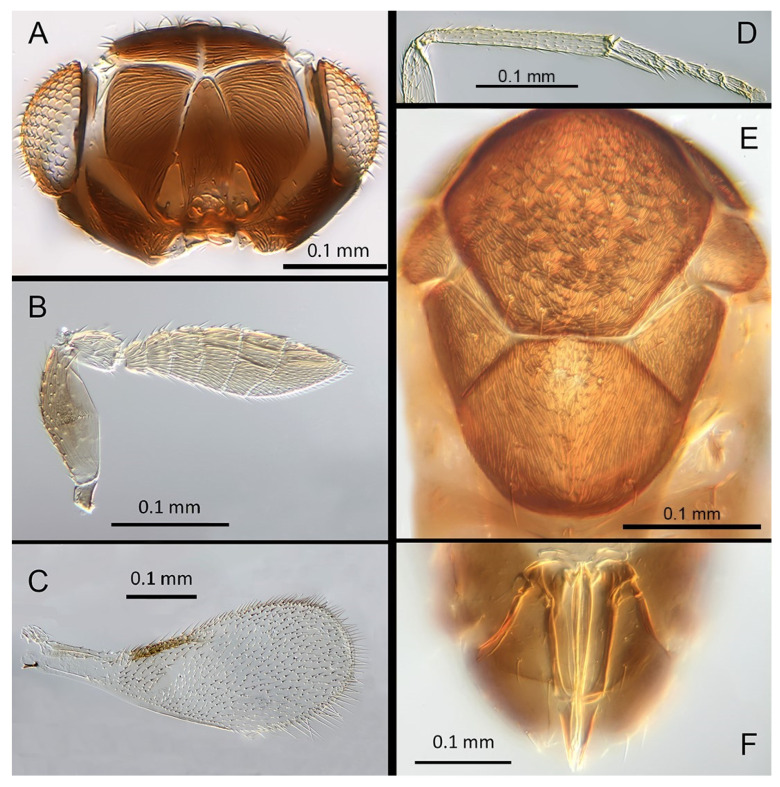
***Encarsia mendesi***: (**A**) head; (**B**) antenna; (**C**) fore wing; (**D**) mid leg; (**E**) dorsal mesosoma; (**F**) ovipositor.

Morphology: Head (Figure 18A) with mediofrontal line complete check, ocellus; transfacial line evident; facial lines very broad along their entire lengths, especially at level of lower eye and adjacent to genae. Scrobes largely smooth, some irregular sculpture centrally. Antenna (Figure 18B): with funicle apparently absent, so the entire flagellum clavate (antennal formula therefore 1, 1, 6); scape expanded, 2.3–2.8x pedicel length; pedicel 2.4x F1; F1 0.9x F2; F2 0.8x F3; funicle 0.5x clava; F5 and F6 broadly oblique, claval sensorial complex developed. Flagellum with the following number of longitudinal sensilla: F1: 0; F2: 1; F3: 1; F4: 3; F5: 4; F6: 3. Mandibles missing from holotype; paratype (male) apparently with 2 teeth. Maxillary palps two-segmented. Mid-lobe of mesoscutum (Figure 18E) with fewer than 30 setae; each lateral lobe with two setae; each axilla with one seta; scutellum with four setae. Sculpture of mesoscutum transverse. Fore wing (Figure 18C) with two large setae and 2–3 smaller setae on submarginal vein, 3–5 setae in basal cell, 7–11 setae on anterior margin of marginal vein, and one seta at the junction of the submarginal vein and parastigma. Linea clava present. Submarginal equal to marginal vein; maximum width of fore wing 2.56x fore wing width, maximum width of wing 4.6x longest seta on marginal fringe. Ovipositor (Figure 18F) 0.8x mid tibial length; third valvulae 0.3x ovipositor length; second valvifer 2.13x third valvula. Mid tibial spur (Figure 18D) equal to corresponding basitarsus. Metasomal terga T1–T7 with 0, 1 + 1, 1 + 1, 1 + 1, 1 + 2 + 1, 1 + 2 + 1 and six setae, respectively. T7 (Figure 18F) rounded covering ovipositor third valvula.

Male. All aspects of coloration and morphology as for female, except the antennae and genitalic characters.

Distribution: BRAZIL: São Paulo.

Host: Aleurodicinae: *Aleurodicus maritimus* Hempel.

Material examined: Holotype ♀ BRAZIL, São Paulo, Mogi-Guazu, 12.v.1981 (M. Cytrynowicz) 84/8 ex *Aleurodicus maritimus* (NHMUK). Paratype 1♂ BRAZIL, São Paulo, Mogi-Guazu, 12.v.1981 (M. Cytrynowicz) 84/8 ex *Aleurodicus maritimus*. 1♂ 1♀ BRAZIL, São Paulo, E.E. Mogi-Guazu, 12.v.1981 (M. Cytrynowicz) 94 ex *Aleurodicus maritimus* on *Bauhinia holophylla* (Bong.) (Fabaceae) (all NHMUK).

Remarks. Morphologically *E. mendesi* appears closest to *E. marynoyesae* having the entire flagellum more or less clavate. It differs from that species by the very short ovipositor. No molecular data were available for *E. mendesi*.

#### 3.3.16. *Encarsia mexicana* (Howard) **comb. n.**

(Figure 19A–F)

*Mesidia mexicana* Howard, 1907: 74

*Dirphys mexicana* (Howard, 1914): 81

Female. Colour: Antennae pale brown, slightly darker on the base of the scape and radicle. Head dark brown with pale lines bordering the eyes and extending along the genae towards clypeus, antennal scrobes, a line from the apex of the scrobes to the median ocellus, and a transverse line midway between the antennal sockets and the median ocellus centrally bordering the dorsal end of antennal scrobes. Mesosoma and metasoma dark brown, with the posterior two-thirds of the scutellum and sides of the metanotum yellow. Legs yellow, with mid and hind coxae and femora partly brown. Wings slightly infuscated below the marginal vein; submarginal, marginal and stigmal veins dark. 

**Figure 19:**
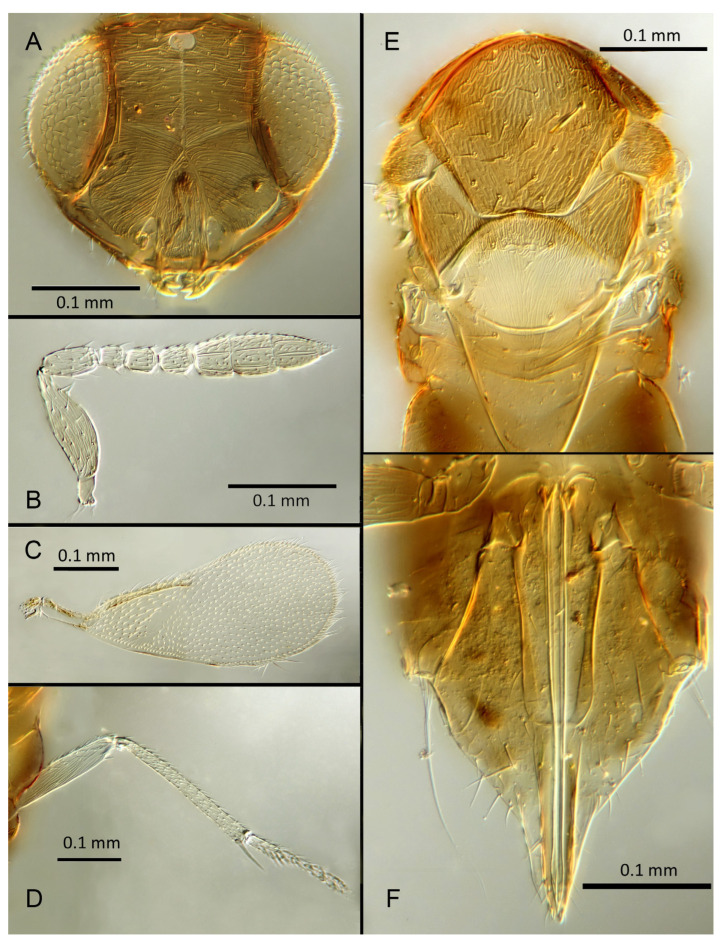
***Encarsia mexicana***: (**A**) head; (**B**) antenna; (**C**) fore wing; (**D**) mid leg; (**E**) dorsal mesosoma; (**F**) ovipositor.

Morphology: Head (Figure 19A) with mediofrontal line complete; transfacial line narrow; facial lines very broad adjacent to genae. Scrobes almost entirely with longitudinal sculpture. Antennal formula (Figure 19B): 1, 1, 3, 3; scape slightly expanded, 2.45x pedicel length; pedicel 1.9x F1; F1 0.8X F2; F2 0.9x F3; funicle 0.57X clava length; F6 slightly oblique, claval sensorial complex developed. Flagellum with the following numbers of longitudinal sensilla: F1: 0; F2: 1; F3: 1; F4: 3; F5: 3–4; F6: 4–5. Mandibles (Figure 19A) with two teeth and a truncation. Maxillary palps two-segmented. Mid-lobe of mesoscutum (Figure 19E) with 30–40 setae; each lateral lobe with two setae; each axilla with one seta; scutellum with four setae. Sculpture of mesoscutum aciculate; sculpture of axillae and scutellum longitudinal. Fore wing (Figure 19C) with two large setae and 3–4 smaller setae on submarginal vein, 3–7 setae in basal cell, 7–10 setae on anterior margin of marginal vein, and one seta at the junction of the submarginal vein and parastigma. Linea calva present. Submarginal 0.85x marginal vein. Maximum length of fore wing 2.7x fore wing width, maximum width of wing 4.65x longest setae on marginal fringe. Ovipositor (Figure 19F) length 1.3x mid tibial length; third valvula 0.5x ovipositor; second valvifer 1.3x third valvula; Mid tibial spur (Figure 19D) 0.9x corresponding basitarsus. Metasomal terga T1–T7 with 0, 2 + 2, 2 + 2, 2 + 2, 1 + 2 + 1, 1 + 2 + 1 and four setae, respectively. T7 (Figure 19F) elongate covering third valvula of ovipositor.

Distribution: COSTA RICA: Limon, Heredia, Arenal, Alajuela; MEXICO: Tabasco.

Host: Nealeurodicus altissimus (Quaintance) (=Ceraleurodicus altissimus).

Material examined: Holotype. MEXICO, Tabasco, San Francisco del Peal. 1.vii.1887 (C.H. Townsend) (USNM). Examined. 6♀ [compared with type series AP xi.90] MEXICO, Tabasco, San Francisco del Peal, 1.vii.1897 (C.H.T. Townsend) ex *Nealeurodicus altissimus* [reared from *Lippia myriocephala*] (NHMUK, USNM). 1♀ COSTA RICA, Heredia, Est. Biol. La Selva, 27–28.ii.2003 (J.S. Noyes) 75 m 10°26′ N 84°01′ W. [DNA 144: OQ683560] (NHMUK); 1♀ COSTA RICA, Arenal, Sen. Pilon, 26.ii.2003 (J.S. Noyes) [DNA 52] (NHMUK); 1♀ COSTA RICA, Alajuela, Est. Caribe R. Rincón Forestal, 19–20.ii.2003 (J.S. Noyes) 400 m, 10°53′ N 85°18′ W [DNA 166] (NHMUK); 1♀ COSTA RICA, Alajuela, P.N. Arenal, send. Pilon, 26.ii.2003 (J.S. Noyes), 600 m 10°27′ N 84°43′ W; [DNA 129] (MZUCR).

Remarks: *Encarsia mexicana* is morphologically closest to *E. fredbennetti* from which it differs by the non-enlarged clava. It is also molecularly closest to *E. fredbennetti*. Sequence data deposited at GenBank accession number: OQ683560.

#### 3.3.17. *Encarsia napo* Polaszek and Hernández-Suárez **sp. n.**

(Figure 20A–F)

Female. Colour: Antennae pale brown, slightly darker on the base of the clava, scape, pedicel, and radicle. Head dark brown. Mesosoma and metasoma uniformly dark brown with the posterior two-thirds of the scutellum and sides of the metanotum yellow. Legs yellow, with hind femur, posterior half of mid femur and anterior half of fore femur light brown, all tarsi pale. Wings hyaline, submarginal vein pale in contrast with darker marginal and stigmal veins.

**Figure 20:**
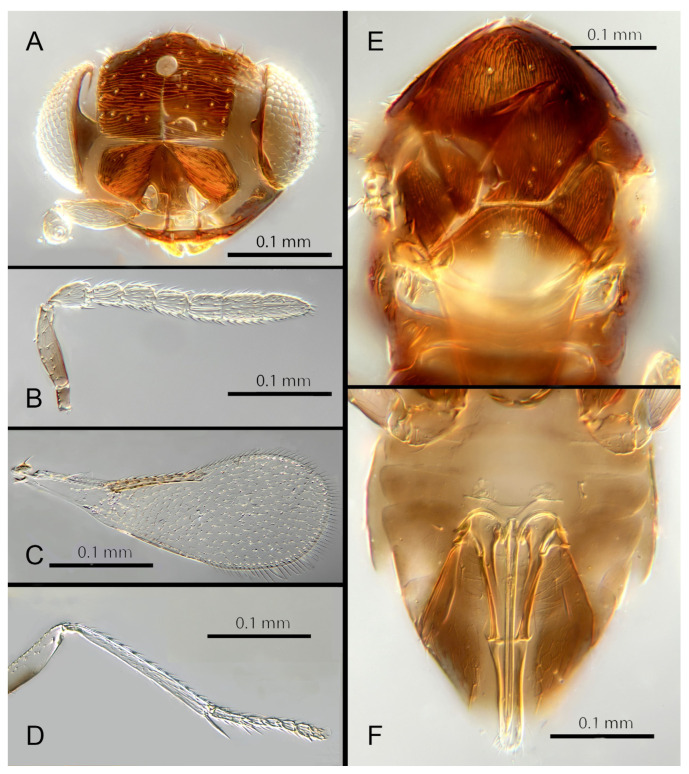
***Encarsia napo***: (**A**) head; (**B**) antenna; (**C**) fore wing; (**D**) mid leg; (**E**) dorsal mesosoma; (**F**) ovipositor.

Morphology: Head (Figure 20A) with mediofrontal line complete; transfacial line evident, broad laterally and tapering towards the middle; facial lines extremely broad along their entire lengths. Scrobes largely smooth, some irregular sculpture centrally. Antennal formula (Figure 19B): 1, 1, 3, 3; scape 2.2x pedicel length; pedicel 1.3x F1; F1 0.88x F2; F2 equal to F3; funicle 0.7x clava; F6 perpendicular. Mandibles (Figure 20A) with two minute teeth and a truncation. Flagellum with the following number of longitudinal sensilla: F1: 1; F2:1; F3: 1 F4: 2 F5: 3; F6: 3. Maxillary palps two-segmented. Mid-lobe of mesoscutum (Figure 20E) with fewer than 20 setae; each lateral lobe with two setae; each axilla with one seta; scutellum with four setae and two vestigial setal bases. Sculpture of mesoscutum, axillae and scutellum longitudinal. Fore wing with two large setae and four smaller setae on submarginal vein, 4–5 setae in basal cell, 7–8 setae on anterior margin of marginal vein, and one seta at the junction of the submarginal vein and parastigma. Linea calva absent. Submarginal 0.80x times marginal vein. Maximum length of fore wing 2.68x fore wing width, maximum width of wing 4.79x longest setae on marginal fringe. Ovipositor (Figure 20F) 0.8x mid tibial length; third valvulae 0.45x ovipositor length; second valvifer 1.2x third valvula. Mid tibial spur (Figure 20D) 0.9x corresponding basitarsus. Metasomal terga T1–T7 with 0, 1 + 1, 1 + 1, 1 + 1, 1 + 2 + 1, 1 + 2 + 1 and six setae, respectively. T7 (Figure 20F) rounded, not extended but covering ovipositor.

Distribution: ECUADOR: Napo River.

Material examined: Holotype ♀ ECUADOR, Napo, transect Ent. 1 km S Onkone Gare Camp, Res. Etnica Waorani, 220 m 0°39′10″ S 76°26′00″ W T.L. Erwin et al. fogging tf forest lot 1193 5.v.1995 [DNA314] (NHMUK). Paratypes 2 slides ♀ ECUADOR, Napo, transect Ent. 1 km S Onkone Gare Camp, Res. Etnica Waorani, 220 m 0°39′10″ S 76°26′00″ W T.L. Erwin et al. fogging tf forest lot 1193 5.v.1995 [DNA 312, 313] (NHMUK). 

Remarks: *Encarsia napo* is morphologically closest to *E. erwini* from which it differs by the partly pale mesoscutellum. No molecular data were available for *E. napo*.

Etymology: Named for the Napo River (Rio Napo) on which the type locality is located.

#### 3.3.18. *Encarsia noora* Polaszek and Hernández-Suárez **sp. n.**

(Figure 21A–E)

Female. Colour: Antennae pale yellow. Head brown with pale lines bordering the eyes and extending along the genae towards clypeus. Mesosoma brown but with posterior three-quarters of scutellum pale. Metasoma uniformly light brown. Legs pale yellow; hind coxae and femora brown. Wings hyaline, slightly infuscated below marginal vein; stigmal vein pale in contrast with a darker marginal vein.

**Figure 21:**
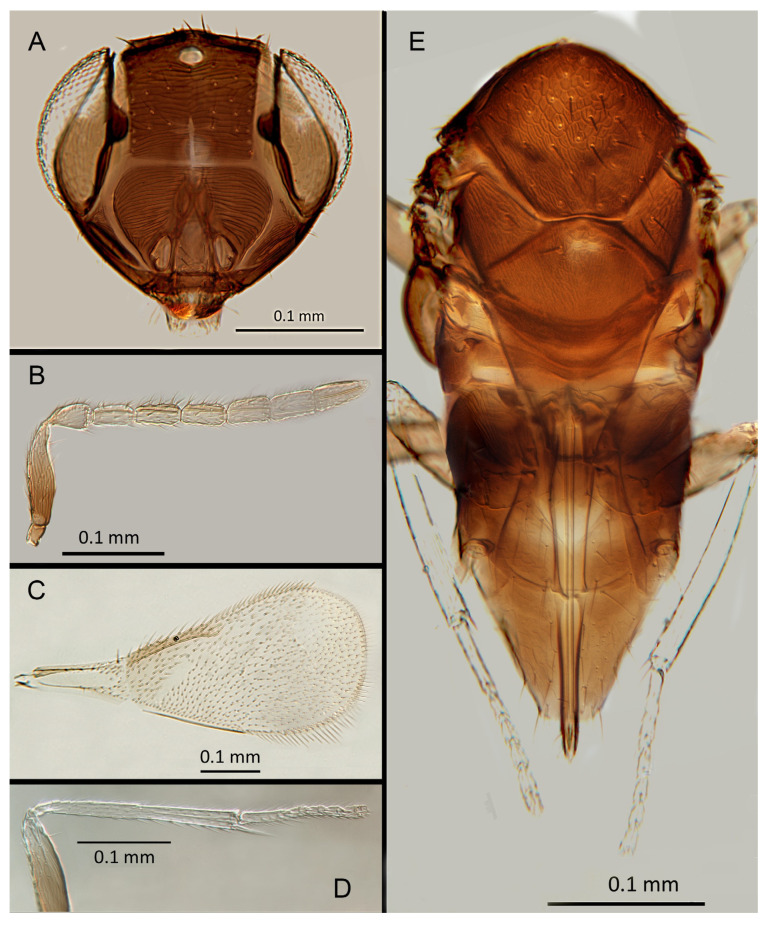
***Encarsia noora***: (**A**) head; (**B**) antenna; (**C**) fore wing; (**D**) mid leg; (**E**) dorsal habitus.

Morphology: Head (Figure 21A) with mediofrontal line incomplete, reaching less than halfway to frontal ocellus, transfacial line evident; facial lines very broad along their entire lengths. Scrobes almost entirely smooth, some irregular transverse sculpture basally. Antennal formula (Figure 21B): 1, 1, 3, 3; scape 2x pedicel length; pedicel 2.2x F1; F1 1.2x F2; F2 0.7x F3; funicle 0.6x clava; F6 slightly oblique, claval sensorial complex apparently present. Flagellum with the following number of longitudinal sensilla: F1: 0; F2: 0; F3: 1–2; F4: 3; F5: 4; F6: 4. Mandibles (Figure 21A) with two min teeth and a truncation. Maxillary palps two-segmented. Mid-lobe of mesoscutum (Figure 21E) with fewer than 30 setae; each lateral lobe with two setae; each axilla with one seta; scutellum with four setae. Sculpture of mesoscutum aciculate; sculpture of axillae and scutellum longitudinal. Fore wing (Figure 21C) with two large setae and four smaller setae on submarginal vein, eight setae in basal cell, 7–8 setae on anterior margin of marginal vein, and one seta at the junction of the submarginal vein and parastigma; a group of long setae below marginal vein. Linea calva present. Submarginal 0.8x marginal vein. Maximum length of fore wing 2.58x fore wing width, maximum width of wing 6.39x longest setae on marginal fringe. Ovipositor (Figure 21E) 1.34x mid tibial length; third valvulae 0.43x ovipositor length; second valvifer 1.2x third valvula. Mid tibial spur (Figure 21D) 0.9x corresponding basitarsus. Metasomal terga T1–T7 with 0, 2 + 2, 2 + 2, 2 + 2, 1 + 2 + 1, 1 + 2 + 1 and four setae, respectively. T7 (Figure 21E) extended although apparently not covering ovipositor.

Distribution: TRINIDAD: Mt St Benedict, St Augustine.

Material examined: Holotype ♀ TRINIDAD, I.C.T.A. [Imperial College of Tropical Agriculture, St. Augustine] xii.1953 (F.D. Bennett) [s9] ex whitefly on cocoa. ?*Coccophagus* sp. Det. FDB [identified as *Coccophagus*, F.D. Bennett] Imperial Parasite Service (NHMUK). Paratypes 2♀ TRINIDAD, Mt. St. Benedict, 18.x.1997 (M. Jagroep) [DNA 126: OQ683561] ex whitefly; [DNA 215b] ex Aleurodicinae (NHMUK).

Remarks: *Encarsia noora* is morphologically closest to *E. catula*, differing by the longer F1 in *E. noora*. Molecularly sister species to *E. aisha*. Sequence deposited at GenBank accession number: OQ683561.

Etymology: Named for Noora, daughter of the second author (EHS), and sister to Aisha; see *E. aisha*, above.

#### 3.3.19. *Encarsia svetlana* Polaszek and Hernández-Suárez **sp. n.**

(Figure 22A–F)

Female. Colour: Antennae light brown slightly darker on F6 and radicle. Head brown with pale lines bordering the eyes and extending along the genae towards clypeus. Mesosoma dark brown but with scutellum and sides of the metanotum yellow. Metasoma light brown. Legs uniformly pale yellow. Wings infuscated below marginal vein, stigmal vein pale in contrast with a darker marginal vein.

**Figure 22:**
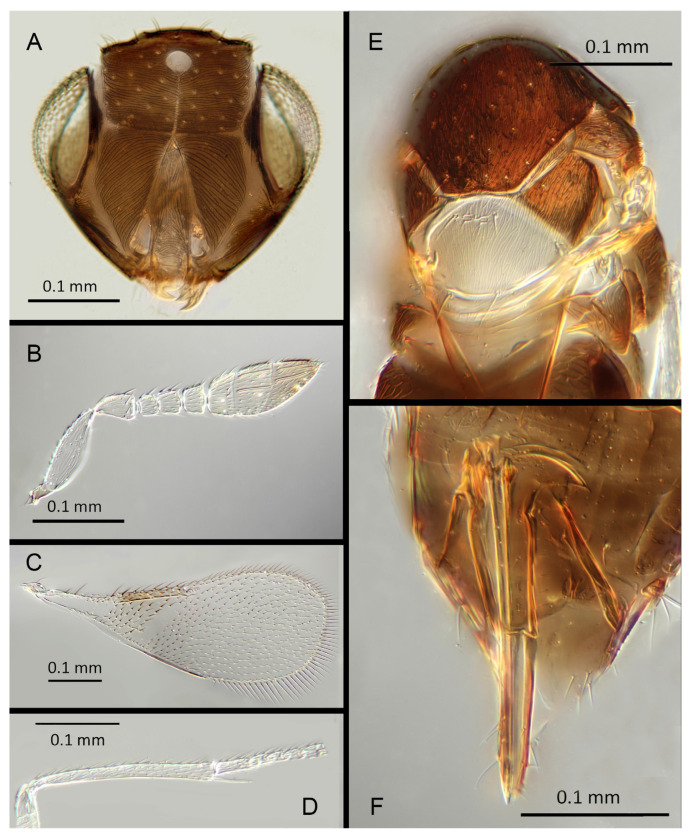
***Encarsia svetlana***: (**A**) head; (**B**) antenna; (**C**) fore wing; (**D**) mid leg; (**E**) dorsal mesosoma; (**F**) ovipositor.

Morphology: Head (Figure 22A) with mediofrontal line complete; transfacial line narrow; facial lines very broad at level of lower eyes and adjacent to genae. Scrobes with longitudinal aciculate sculpture in the upper half, transverse aciculate sculpture in the lower half. Antennal formula (Figure 22B): 1, 1, 3, 3; scape 2.25x pedicel length; pedicel 2.36x F1; F1 0.86x F2; F2 0.9x F3; funicle 0.49x clava length; F6 broadly oblique, claval sensorial complex developed. Flagellum with the following number of longitudinal sensilla: F1: 0; F2: 1; F3: 1; F4: 1; F5: 4; F6: 4. Mandibles (Figure 22A) with 2 very large ventral teeth and a small truncation, appearing bidentate. Maxillary palps 2-segmented. Mid-lobe of mesoscutum (Figure 22E) with fewer than 30 setae; each lateral lobe with two setae; each axilla with one seta; scutellum with four setae. Sculpture of mesoscutum, axillae and scutellum longitudinal. Fore wing (Figure 22C) with two large setae and four smaller setae on submarginal vein, four setae in basal cell, 6–7 setae on anterior margin of marginal vein, and one seta at the junction of the submarginal vein and parastigma. Linea calva present. Submarginal 0.9x marginal vein. Maximum length of fore wing 2.5x fore wing width, maximum width of wing 3.85x longest setae on marginal fringe. Ovipositor (Figure 22F) 0.6x mid tibial length; third valvulae 0.95x ovipositor length; second valvifer 1.1x third valvula. Mid tibial spur (Figure 22D) 0.9x corresponding basitarsus. Metasomal terga T1–T7 with 0, 2 + 2, 2 + 2, 1 + 1, 1 + 1 + 1, 1 + 1 and four setae, respectively. T7 (Figure 22F) apex rounded not covering ovipositor.

Distribution: GUYANA: Dubulay Ranch.

Material examined: Holotype ♀ GUYANA, Dubulay Ranch, 17–22.i.1999 (M. Sharkey and B. Brown) [DNA 305: OQ683558] Univ. Calif. Riverside, Ent. Res. Museum UCR ENT 182858 5°40.954′ N 57°51.524′ W (UCRC).

Remarks: *Encarsia svetlana* is morphologically closest to *E. venia* from which it differs in having an F3 transverse, and its scape is entirely pale. Sequence data deposited under GenBank accession number: OQ683558. 

Etymology: Named for Svetlana Myartseva, prolific describer of Mexican Aphelinidae, including many *Encarsia* species.

#### 3.3.20. *Encarsia venia* Polaszek and Hernández-Suárez **sp. n.**

(Figure 23A–F)

Female. Colour: Antennae light brown, slightly darker on scape and radicle. Head dark brown. Mesosoma and metasoma dark brown, with entire scutellum and sides of the metanotum yellow. Legs uniformly pale yellow. Wings hyaline, slightly infuscated below marginal vein.

**Figure 23:**
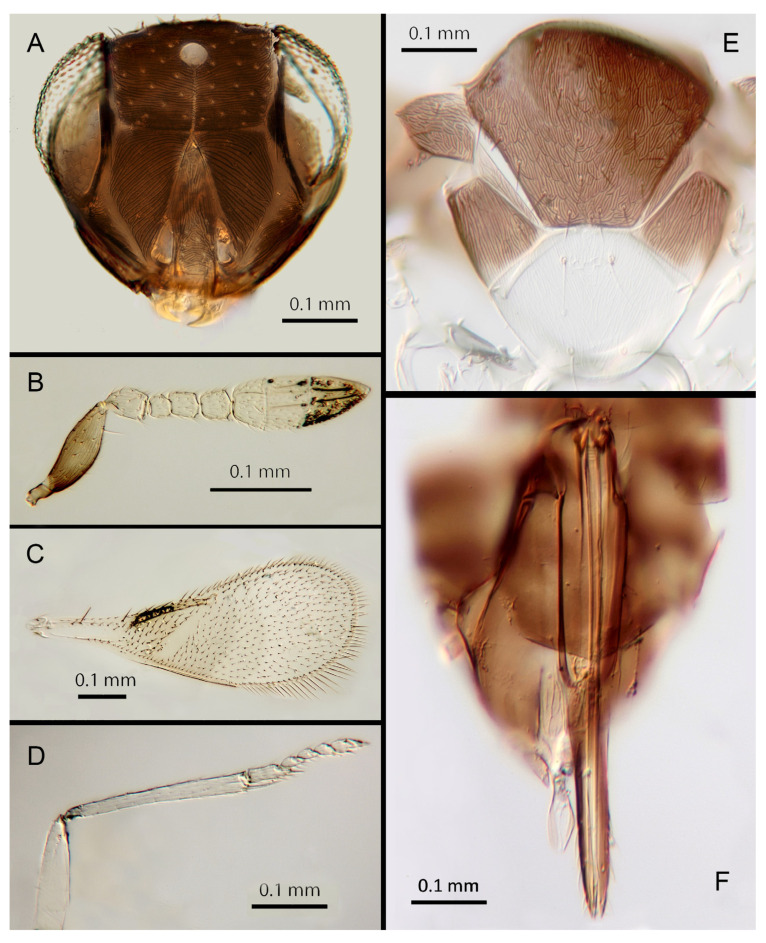
***Encarsia venia***: (**A**) head; (**B**) antenna; (**C**) fore wing; (**D**) mid leg; (**E**) dorsal mesosoma; (**F**) ovipositor.

Morphology: Head (Figure 23A) with mediofrontal line complete, very narrow towards frontal ocellus; transfacial line narrow; facial lines broad along their entire lengths. Scrobes with longitudinal aciculate sculpture in the upper half, transverse aciculate sculpture in the lower half. Antennal formula (Figure 23B): 1, 1, 3, 3; scape 2.4x pedicel length; pedicel 2x F1; F1 0.7x F2; F2 1.1x F3; funicle 0.6x clava; F6 slightly oblique, claval sensorial complex apparently present. Flagellum with the following number of longitudinal sensilla: F1: 0; F2: 1; F3: 1; F4: 2; F5: 3; F6: 3. Mandibles (Figure 23A) with one very large ventral tooth and an upper bidentate tooth. Maxillary palps two-segmented. Mid-lobe of mesoscutum (Figure 23E) with fewer than 30 setae; each lateral lobe with two setae; each axilla with 1 seta; scutellum with four setae. Sculpture of mesoscutum aciculate; sculpture of axillae and scutellum longitudinal. Fore wing with two large setae and four smaller setae on submarginal vein, 3–4 setae in basal cell, 7–8 setae on anterior margin of marginal vein, and one seta at the junction of the submarginal vein and parastigma. Linea calva present. Submarginal equal to marginal vein. Maximum length of fore wing 2.8x fore wing width, maximum width of wing 4.5x longest setae on marginal fringe. Ovipositor (Figure 23F) 1.4x mid tibial length; third valvulae 0.4x ovipositor length; second valvifer 1.3x third valvula. Mid tibial (Figure 23D) spur equal to corresponding basitarsus. Metasomal terga T1–T7 with 0, 1 + 1, 1 + 1, 2 + 2, 1 + 2 + 1, 1 + 2 + 1 and four setae, respectively. T7 (Figure 23F) apex extended but not covering ovipositor entirely.

Distribution: COSTA RICA: Heredia.

Material examined: Holotype ♀ COSTA RICA, Limon, Parque Nacional Cahuita 2 m, 26.ii.2004 (J.S. Noyes) [DNA 298: OQ683557] 9°43′ N 82°49′ W (NHMUK). 1♀ COSTA RICA, Limon, Hitoy-Cerere, 21–22.iii.2006 (J.S. Noyes) [DNA 267] 90°40′ N 83°02′ W (NHMUK). 1♀ COSTA RICA, Heredia La Selva, 22.i–2.ii. 1991 (J.S. Noyes).

Remarks: Morphologically closest to *E. svetlana* from which it differs in having F3 quadrate (transverse in *E. svetlana* and a dark scape. Sequence deposited under GenBank accession number: OQ683557.

Etymology: From Latin *venia* meaning “kindness”.

#### 3.3.21. *Encarsia myartsevae* Kresslein and Polaszek **nom. nov.**

Remarks: Myartseva (2007) originally described this species as *Encarsia mexicana* Myartseva. In synonymizing *Dirphys* and *Encarsia*, this species name becomes preoccupied by the new combination *Encarsia mexicana* (Howard, 1907). As a result, we rename this species *Encarsia myartsevae* **nom. nov.** in honor of the species’ author, Svetlana Myartseva, for her contributions to the systematics of *Encarsia* and in particular her dedication to providing accounts of their host associations. *Encarsia myartsevae* **nom. nov.** is not a member of the *Encarsia mexicana* species-group, instead belonging to the *opulenta* species-group, and is not discussed further in this revision.

### 3.4. Key to the species of the Encarsia mexicana species-group: Females

1. Linea calva absent (Figure 9C)………………………………………………………………………………………………………2
-Linea calva present (Figure 4E)………………………………………………………………………………………………………7
2. Two setae on axilla (Figure 11E)………………………………………………………………………………………….*E. dichaeta*

-One seta on axilla (Figure 4C)………………………………………………………………………………………………………..3
3. Mesoscutum with transverse sculpture (Figure 10E)…………………………………………………………………*E. diablejo* *
-Mesoscutum with aciculate (Figure 9E) or longitudinal (e.g., Figure 12E) sculpture…………………………………………4
4. Mid lobe of mesoscutum with more than 30 setae (Figure 9E)…………………………………………………….
*E. cylindrica*

-Mid lobe of mesoscutum with fewer than 30 setae (e.g., Figure 12E)…………………………………………………………..5
5. Third valvulae longer than second valvifers (Figure 12F)………………………………………………………..
*E. encantadora*

-Thirds valvulae shorter than second valvifers (e.g., Figure 13F)………………………………………………………………..6
6. Mesoscutellum entirely dark (Figure 13E); hind femora pale…………………………………………………………
*E. erwini*

-Mesoscutellum pale in lower half (Figure 20E); hind femora dark……………………………………………………...*E. napo*
7. Mid lobe of mesoscutum with fewer than 30 setae (e.g., Figure 18E)………………………………………………………….8
-Mid lobe of mesoscutum with more than 30 setae (e.g., Figure 17E)………………………………………………………….12
8. Ovipositor shorter than mid tibia……………………………………………………………………………………….
*E. mendesi*

-Ovipositor longer than mid tibia……………………………………………………………………………………………………9
9. Mesoscutellum entirely pale (e.g., Figure 22E)………………………………………………………………………………….10
-Mesoscutellum entirely dark (e.g., Figure 8E)……………………………………………………………………………………11
10. F3 wider than long; scape pale (Figure 22B)………………………………………………………………………….
*E. svetlana*

-F3 quadrate; scape dark (Figure 23B)………………………………………………………………………………………. *E. venia*
11. F1 shorter than, or equal to, F2 (Figure 8B) …………………………………………………………………………….
*E. catula*

-F1 longer than F2 (Figure 21B)……………………………………………………………………………………………… *E. noora*
12. F1 equal in length to F2, or longer ………………………………………………………………………………………………13
-F1 shorter than F2……………………………………………………………………………………………………………………15
13. Suture between F5 and F6 oblique (e.g., Figure 17B)………………………………………………………………………….14
-Suture between F5 and F6 perpendicular (Figure 16B)…………………………………………………………………*E. larensis*
14. Second valvifers almost 2x length third valvulae (Figure 17F)………………………………………………..
*E. marynoyesae*

-Second valvifers about 1.5x length third valvulae (Figure 5F)………………………………………………………….. *E. aisha*
15. Mesoscutellum entirely dark……………………………………………………………………………………………
*E. aphania*

-Mesoscutellum at least partly pale (e.g., Figure 14C)…………………………………………………………………………..16
16. Ovipositor more than 1.8x mid tibia (Figure 4B)……………………………………………………………………….
*E. acusa*

-Ovipositor less than 1.6x mid tibia………………………………………………………………………………………………..17
17. Fore wing infuscate posterior to the marginal vein, and with robust setae proximad linea calva (e.g., Figure 15B)…18
-Fore wing lacking the above characters states…………………………………………………………………………………..19
18. Antenna with clava enlarged; 3.5x longer than wide (Figure 14D)…………………………………………..
*E. fredbennetti*

-Antenna with clava not enlarged; 2.3x longer than wide (Figure 15C)……………………………………………….*E. inbioa*
19. Antenna with scape and F6 dark (Figure 7B)…………………………………………………………………………..
*E. avida*

-Antenna with scape and F6 pale (Figure 19B)………………………………………………………………………..*E. mexicana*

* Unknown female of *E. diablejo* assumed to share mesosomal sculpture with the male. An accompanying multiple entry key can be found at: https://rkres001.github.io/rkres001.encarsiamexicanakey/ (accessed on 6 June 2023).

## 4. Discussion

All taxa of *Encarsia mexicana* species-group recovered sister to *Encarsia dichaeta* have a linea calva present on the fore wing (absent in *E. dichaeta*; unknown D2672, and DNA.0165). The presence or absence of a linea clava may represent a local synapomorphy for these two clades within the *mexicana*-group. All taxa which lack a linea calva also lack the enlargement of the clava of the antenna common in this species-group, though *Encarsia noora* possesses a linea calva in the absence of an enlarged clava. Further phylogenetic analyses and sequence capture for the five other species without linea calva (*E. diablejo*, *E. cylindrica*, *E. encantadora*, *E. erwini* and *E. napo*) will be necessary to determine the informativeness of these characters. Other evident characters appear phylogenetically uninformative. Some surprises include *E. svetlana* appearing well-removed from *E. venia* while their morphology is extremely similar. *E. aisha* and *E. noora* despite being DNA sister species have markedly different flagellar shapes.

With the placement of *Dirphys* **syn. n.** in the middle of *Encarsia*, the genus expands to 473 described species. Establishment of the *Encarsia mexicana* species-group further increases the difficulty with which the genus can be recognized. In particular, the genus now contains species with a linea calva on the fore wing, and parasitoids with a gregarious life history, further expanding the already broad host range of the genus. The future classification of *Encarsia* at large will rely upon its clarification by robust phylogenetic hypotheses built upon large molecular datasets (Kresslein et al. unpublished). As the phylogeny of *Encarsia* is further resolved, it will be necessary to revisit the current classification of the genus and determine whether alternative generic classifications would allow for greater diagnosability of the taxa therein.

## Figures and Tables

**Table 1:** Ingroup taxa (species identity, voucher IDs, accession numbers, locality, plant associate and host).

Species	Dna Code	Accesion Number	Locality	Plant Associate	Host(s)
*Encarsia acusa*	DNA0148	OQ683554	Costa Rica: Heredia, Est. Biol. La Selva		
	DNA0212		Peru: Loreto, Iquitos, Barillal		
*Encarsia aisha*	DNA0146	OQ683562	Costa Rica: Heredia, Est. Biol. La Selva		
	DNA0164	OQ683562	Costa Rica: Alajuela, Est. Caribe		
*Encarsia aphania*	DNA0218	OQ683546	Belize: Cayo, Las Cuevas		*Aleurodicus pulvinatus*
			Belize: Cayo, Las Cuevas		*Azuraleurodicus pentarthus*
			Belize: Cayo, Chiquibul	*Inga* sp.	*Nealeurodicus altissimus*
	DNA0213	OQ683545	Costa Rica: Puntarenas		
*Encarsia avida*	DNA0143	OQ683547	Costa Rica: Heredia, Est. Biol. La Selva		
*Encarsia catula*	DNA0278	OQ683547	Costa Rica: Limon, Hitoi-Cerere		
*Encarsia cylindrica*			Brazil: Minas Gerais, Vicosa,	*Citrus* sp.	*Aleurothrixus floccosus (?)*
	DNA0209		Costa Rica: Puntarenas RF, Piedras Blancas		
	DNA0211		Costa Rica: San Juan, Ciudad Colon		
	DNA0149		Costa Rica: Heredia, Est. Biol. La Selva		
			Jamaica: Fair Prospect		*Aleurodicus jamaicensis*
*Encarsia diablejo*			Peru: Loreto, Iquitos		
*Encarsia dichaeta*			Brazil: Bahia		*Aleurodicus flavus*
	DNA0132-0135	OQ683550	Costa Rica: Alajuela, P.N. Arenal, Pilon		
	DNA0133-0136	OQ683552	Costa Rica: Alajuela, P.N. Arenal, Pilon		
	DNA0208	OQ683551	Costa Rica: Guanacaste, Pitilla		
	DNA0151	OQ683549	Costa Rica: Heredia, Est. Biol. La Selva		
			Ecuador: Napo, Anangucocha		*Aleurodicus* sp.
*Encarsia encantadora*			Ecuador: Napo River		
			Mexico: Tabasco	*Lippia myriocephala*	*Nealeurodicus altissimus*
*Encarsia erwini*			Ecuador: Napo River		
*Encarsia fredbennetti*	DNA0215B	OQ683559	Trinidad and Tobago: Trinidad, St Augustine	*Theobroma cacao*	Aleurodicinae
	DNA0216		Trinidad and Tobago: Trinidad, Mount St Benedict		
*Encarsia inbioa*	DNA0128	OQ683553	Costa Rica: Alajuela, P.N. Arenal, Pilon		
*Encarsia larensis*			Venezuela: Cabudare, Lara	*Hura crepitans*	*Aleurodicus* *pulvinatus*
*Encarsia marynoyesae*	DNA0163	OQ683563	Costa Rica: Alajuela, Est. Caribe		
	DNA0167		Costa Rica: Alajuela, Est. Caribe		
*Encarsia mendesi*			Brazil: São Paolo, Mogi-Guazu	*Bauhinia holophylla*	*Aleurodicus maritimus*
*Encarsia mexicana*			Mexico: Tabasco, San Francisco del Peal	*Lippia myriocephala*	*Nealeurodicus altissimus*
	DNA0144	OQ683560	Costa Rica: Heredia, Est. Biol. La Selva		
			Costa Rica: Limon		
	DNA0166		Costa Rica: Alajuela, Est. Caribe R.		
	DNA0129		Costa Rica: Alajuela, P.N. Arenal, Pilon		
*Encarsia napo*			Ecuador: Napo River, Camp. Res. Waorani		
*Encarsia noora*	DNA0126	OQ683561	Costa Rica: Limon		
*Encarsia svetlana*	DNA0305	OQ683558	Guyana: Dubulay Ranch		
*Encarsia venia*	DNA0298	OQ683557	Costa Rica: Limon, Parque Nacional Cahuita		
	DNA0267		Costa Rica: Limon, Hitoy-Cerere		
			Costa Rica: Heredia La Selva		
*E. mexicana*-group sp.	DNA0165	OQ683556	Costa Rica: CR Alajuela Est. Caribe R. Rincon Forestal		
	D2672	OQ683555	Ecuador: Orellana, Tiputini Biodiversity Sta.		

**Table 2:** Outgroup taxa (species identity, voucher IDs, accession numbers, and locality).

Species	DNA Code	Accesion Number	Locality
*Encarsia luteola*	D0243	AF223369	USA: California, Brawley
*Encarsia formosa*	D0231	AF223372	Egypt
*Encarsia cubensis*	DNA270	OQ683567	Costa Rica: Limon
*Encarsia azimi*	DNA259P17	AF254229	Australia: Queensland
*Encarsia inaron*	D0465	AY599399	New Zealand
*Encarsia lounsburyi*	DNA017	OQ683568	Costa Rica: Puntarenas
*Encarsia citrina*	DNA376	OQ683569	U.K.: London, Barnes Common
*Encarsia boswelli*	DNAAE534	OQ683570	India
*Encarsia perplexa*	D0296	AF254243	Guatemala: Coatepeque
*Encarsia opulenta*	DNA387	OQ683571	Mexico: Los Tuxtlas
*Encarsia lutea*	D0235	AF254238	Cyprus
*E. noyesi* group sp.	DNA0091	OQ683566	Costa Rica
*Encarsia tamaulipeca*	DNA0123	OQ683564	Ecuador
*E. noyesi* group sp.	DNA0089	OQ683565	Ecuador: Napo
*Encarsia sophia*	D0219	AF254198	Find in Heraty Lab
*Encarsia protransvena*	D0136	AF254208	USA: California, Orange Co.
*Coccophagus* sp.	DNA010	OQ683572	Costa Rica
*Coccophagus* sp.	DNA0185	OQ683573	Costa Rica: La Selva
*Coccophagus lycimnia*	DNAA1-006A	OQ683574	Costa Rica
*Coccophagus semicircularis*	DNAA3-023D	OQ683575	Costa Rica: Puntarenas
*Coccophagus* sp.	DNA034	OQ683576	Costa Rica: Puntarenas
*Aphytis yanonensis*	D0446	AY635336	UCR Culture: Originally from Japan, Fukuoka
*Aphytis melinus*	D0445	AY635342	UCR Culture: Originally from China, Fujian, Fuzhou

## Data Availability

All DNA sequence data that support the results and conclusions of this study can be found at GenBank.
